# A Caged Ret Kinase Inhibitor and its Effect on Motoneuron Development in Zebrafish Embryos

**DOI:** 10.1038/srep13109

**Published:** 2015-08-24

**Authors:** David Bliman, Jesper R. Nilsson, Petronella Kettunen, Joakim Andréasson, Morten Grøtli

**Affiliations:** 1 Department of Chemistry and Molecular Biology, University of Gothenburg, SE-412 96 Gothenburg, Sweden; 2 Department of Chemistry and Chemical Engineering, Physical Chemistry, Chalmers University of Technology, SE-412 96 Gothenburg, Sweden; 3 Institute of Neuroscience and Physiology, Sahlgrenska Academy at University of Gothenburg, SE-413 45 Gothenburg, Sweden

## Abstract

Proto-oncogene tyrosine-protein kinase receptor RET is implicated in the development and maintenance of neurons of the central and peripheral nervous systems. Attaching activity-compromising photocleavable groups (*caging*) to inhibitors could allow for external spatiotemporally controlled inhibition using light, potentially providing novel information on how these kinase receptors are involved in cellular processes. Here, caged RET inhibitors were obtained from 3-substituted pyrazolopyrimidine-based compounds by attaching photolabile groups to the exocyclic amino function. The most promising compound displayed excellent inhibitory effect in cell-free, as well as live-cell assays upon decaging. Furthermore, inhibition could be efficiently activated with light *in vivo* in zebrafish embryos and was shown to effect motoneuron development.

Receptor tyrosine kinases and their related signalling pathways are essential for cell development^1^. A convenient way of manipulating these events is by using small-molecule inhibitors, recognized as seminal tools for affecting biological signalling mechanisms as well as for controlling the associated bio-relevant processes. This is due to the non-covalent interactions with target motifs, the ability to tune their physicochemical/biological properties through organic synthesis, and convenient administration. Since kinase signalling pathways involved in cell- and organ development are inherently time- and space-dependent processes, controlling the action of the implicated enzymes in a spatiotemporal fashion would be of tremendous utility. In the caged approach, the activity of a compound is masked by a photolabile group that can be cleaved off *in situ* using light of a specific wavelength, implying that external (photonic) control can be gained over when and where the compound is active. It follows that caged effectors would represent a powerful technique for manipulating biological processes^2^,^3^,^4^,^5^, and this scheme has consequently been used for *in situ* release of for example ATP^6^, neurotransmitters^7^,^8^,^9^,^10^, and phospholipids^11^. Despite the significance and multitude of protein kinase targets, only a few examples of caged kinase inhibitors have been reported^12^,^13^.

Ret, also referred to as RET (REarranged during Transfection; hereafter, ret will refer to the zebrafish ortholog, while RET refers to the human ortholog), is a receptor tyrosine kinase involved in several processes of biological importance, *e.g.*, the development of the central and peripheral nervous systems. Dysregulation of RET has been found in thyroid cancers, including papillary thyroid carcinomas and multiple endocrine neoplasia type 2^14^,^15^,^16^. RET is therefore interesting both from the viewpoint of developmental biology and as a potential target for cancer treatment. Our group has previously developed a small molecule inhibitor of RET – compound **1** (referred to as **7a** in the previous study^17^), with *in vitro* activity in the low nM range, and inhibitory effect on GDNF-induced RET phosphorylation of ERK1/2. Furthermore, the compound displayed an excellent selectivity profile toward RET, with partial inhibition of only six other tyrosine kinases^17^. In a follow up study the effects of **1** (referred to as SPP86 in the follow up study^18^) on RET-induced signalling and proliferation was assessed^18^. Compound **1** inhibited MAPK signalling and proliferation in RET/PTC1 expressing TPC1 but not 8505C or C643 cells, again highlighting the selectivity of the compound. In MCF7 cells, **1** inhibited PI3K/Akt and MAPK signalling and estrogen receptor α (ERα) phosphorylation, all downstream of RET. It was also found to inhibit proliferation to a similar degree as tamoxifen. No cytotoxicity was observed in any of the cell lines used in these experiments.

Here, we report the design, synthesis, and biological evaluation of a caged small-molecule inhibitor of RET in cell-free and live-cell assays, as well as in zebrafish.

## Results and Discussion

### Design and synthesis of caged RET inhibitor

A wide range of photolabile caging groups is described in the literature^3^. The 6-nitroveratroyloxycarbonyl (NVOC) protecting group is one of the most commonly used caging compounds and has been used for N6-protection of purines^19^, structurally similar to **1**. NVOC can be removed at wavelengths longer than 350 nm, *i.e.* wavelengths sufficiently low in energy to avoid extensive cell damage. Moreover, NVOC-caged retinoic acid has been used to study the effect of retinoic acid on the development of zebrafish embryos and it was reported that no effects of the nitrosoaldehyde byproduct formed in the deprotection were observed^20^.

**Figure 1:**
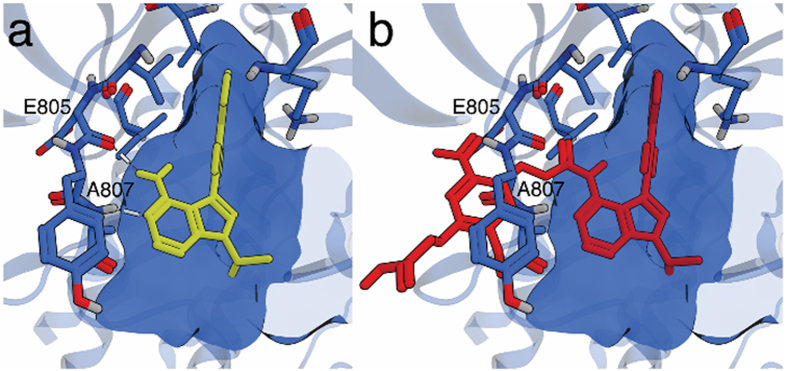
Model of 1 and caged 1 in the ATP-binding site of RET. (**a**) Model of **1** (yellow) docked in the ATP-binding site of RET (blue, pdb: 2IVV) and (**b**) caged **1** (red) superimposed over **1** showing steric clash of the cage and the binding site. Hydrogen bonds between E805, A807 and **1** are represented as white lines.

We chose to protect **1** on the exocylic amino functionality that interacts with the backbone of RET in the ATP-binding site through a hydrogen bond to the amide oxygen of Glu805 (Fig. 1a). A protecting group in this position should substantially lower the affinity of **1** towards RET, both by blocking the hydrogen bond and by introducing steric bulk (Fig. 1b).

**Figure 2:**
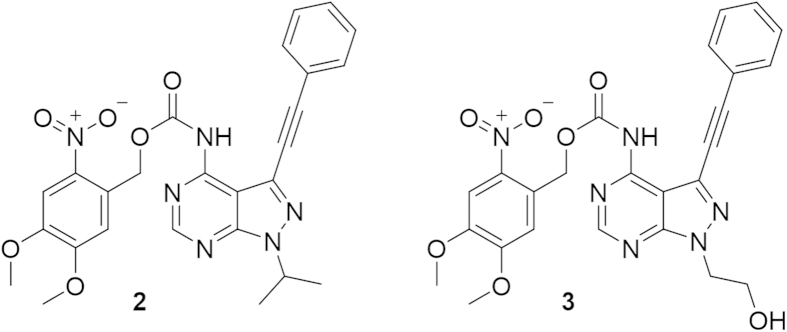
Compounds 2 and 3.

Reacting **1** with 6-nitroveratryloxycarbonyltetrazolide^19^ preformed *in situ* from commercially available 6-nitro-veratrylchloroformate (NVOC-Cl) gave **2** (Fig. 2) in 42% yield (see SI for details). Reacting **1** with NVOC-Cl directly resulted in bisprotected **1** as the main product. Unfortunately, **2** was found to be insufficiently soluble in aqueous media. Introduction of a hydroxyl function on the isopropyl substituent of **1** was expected to increase the hydrophilicity while having a small effect on RET inhibitory activity since this group is located in the sugar binding part of the ATP-binding pocket.

Reacting 1-(2-((*tert-*butyldimethylsilyl)oxy)ethyl)-3-(phenylethynyl)-1H-pyrazolo[3,4-d]pyrimidin-4-amine (see SI for details) with 6-nitroveratryloxycarbonyltetrazolide as described for **1** followed by removal of the silyl protecting group on the hydroxyl function by tetrabutyl ammonium fluoride (TBAF) resulted in **3**. Again, the solubility in aqueous media was too low to be practical for biological assays. The high lipophilicity of the protected compounds necessitated a new approach in which we instead modified the protecting group to increase the hydrophilicity.

**Figure 3:**
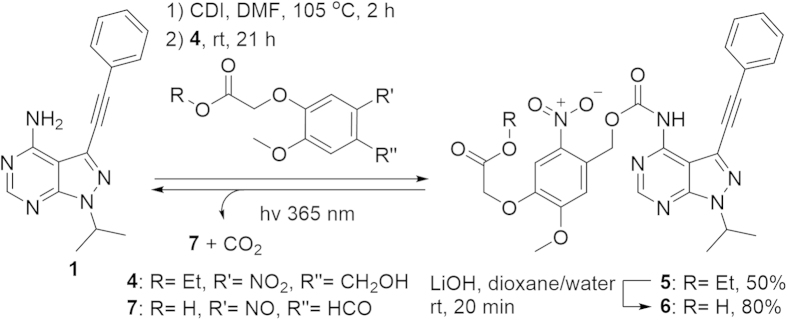
Synthesis and photolysis of 6.

Carboxylic acid analogs of nitrobenzyl protecting groups have been reported^21^,^22^ and should substantially improve the aqueous solubility of the caged compounds. The new protecting group 4-ethyloxycarbonylmethoxy-5-methoxy-2-nitro-benzylalcohol **4** was synthesized from vanillin (14% yield over 3 steps, see SI for details). Using a protocol developed for *t*Boc-protection of primary anilines^23^, **1** was heated at 105 °C with carbonyldiimidazole (CDI) in DMF followed by addition of **4** which resulted in **5** (50% yield, Fig. 3). Hydrolysis of the ethyl ester with LiOH in water and dioxane (1:1) resulted in **6** (80% yield).

Indeed, this compound was soluble in aqueous buffer (1 vol% DMSO) up to 100 μM. Photoinduced cleavage of **6** was readily achieved with 365 nm light, as monitored by HPLC. The decaging followed first order kinetics (τ = 9.6 min, light flux: 1.5 mW/cm^2^, (Fig. S1) with respect to disappearance of **6**, as well as liberation of **1**. As for thermal stability of **6** in aqueous buffer, no changes in the absorption spectrum were detected over 48 h in 37 °C (Fig. S3), indicating excellent stability toward thermal degradation.

### Photocontrolled inhibition of RET

With the caged compound in hand, the ability to photocontrol the inhibition of RET kinase was assessed. For this purpose, an *in vitro* assay with purified RET kinase was used to measure ADP production during substrate phosphorylation. Compound **6** was added to two parallel preparations comprising RET kinase and substrate. One preparation was exposed to light (365 nm, 15 min), while the other was kept in the dark.

**Figure 4:**
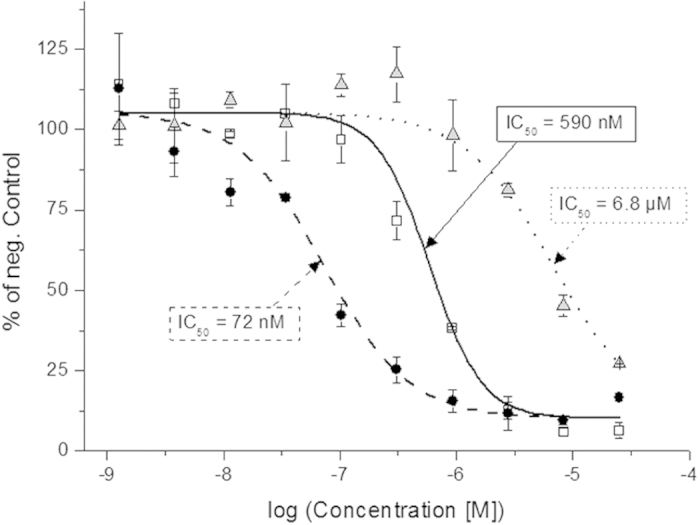
*In vitro* RET incubation. RET-induced ATP turnover was monitored *via* luminescence intensity (see Methods section for activity detection). The activity following incubation with **1** (circles), **6** (triangles) and light-exposed **6** (15 min 365 nm, squares) was referenced to a negative control incubation (without compound added). Fitting to the Hill equation rendered IC_50_ values of 72 nM, 6.8 μM, and 590 nM for **1** (dashed line), **6** (dotted line), and irradiated **6** (solid line), respectively. Data is represented as mean ± standard deviation of duplicate samples.

After adding ATP and incubating at room temperature for 30 min, the relative ATP turnover was assessed. It was shown that the inhibitory capability of **6** increased 12-fold upon light exposure (IC_50_: 6.8 μM → 590 nM), clearly illustrating light-activation of the RET kinase inhibitor (Fig. 4). For example; irradiating an administered dose of 1.6 μM **6**, would modulate the RET kinase activity from 90% to 10%.

The inhibitory effect of **6** observed without irradiation could originate from weak binding of **6** and/or from minute amounts of contamination of free **1** (<0.5% by HPLC, Fig. S23). Dose-response experiments with **1** gave an IC_50_ of 72 nM. The higher IC_50_ measured for the decaged compound was expected, since deprotection was not complete within the applied 15 min of irradiation (Fig. S1). Complete (>99%) decaging would have required at least 45 minutes exposure time. The applied 15 min was chosen as a compromise to balance the risk of UV induced harm with the extent of decaging. An important prerequisite for all experiments involving irradiation is that the investigated system is unaffected by the applied light-dose itself. RET activity was therefore measured in the absence of inhibitor with and without 365 nm irradiation. No changes in kinase activity could be detected after up to 15 min of light exposure (Fig. S2).

**Figure 5:**
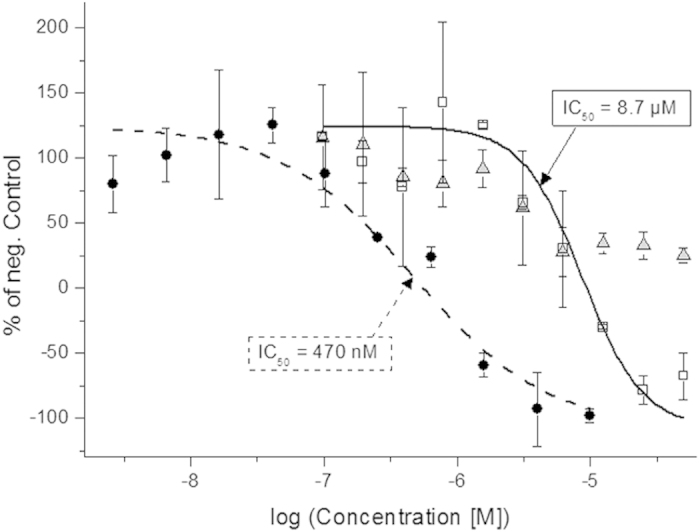
Live-cell RET incubation. RET activity was monitored *via* luminescence intensity (see Methods section for activity detection). The activity following incubation with **1** (circles), **6** (triangles) and light-exposed **6** (15 min 365 nm, squares) was referenced to a negative control incubation (without compound added). Fitting to the Hill equation rendered IC_50_-values of 470 nM and 8.7 μM for **1** (dashed line) and irradiated **6** (solid line), respectively. We were unable to extract meaningful IC_50_-data with non-irradiated **6** included in the fit. Data is represented as mean ± standard deviation of duplicate samples.

The photoactivation of **6** was also tested in a RET functional whole cell assay which utilizes β-galactosidase to produce a luminescent readout^24^,^25^,^26^ (see Methods section for assay details). Compound **6** was added to live cells expressing RET kinase in two identical preparations, followed by 3 h preincubation at 37 °C. At this point, one preparation was exposed to light (365 nm, 15 min), while the other was kept in the dark. Again, 15 min is clearly not sufficient to complete the decaging (see above). Addition of the growth factor neurturin (required to activate the RET kinase) and incubation for 3 h at 22 °C allowed for activity-correlating signal induction using the supplemented detection reagents. In accordance with the cell-free assay, a clear difference in kinase activity was observed after incubation with irradiated (365 nm for 15 min) *vs.* non-irradiated **6**. Irradiated **6** showed an IC_50_ of 8.7 μM (Fig. 5) while non-irradiated **6** displayed only partial inhibition (*ca.* 40%) at concentrations higher than 6 μM. However, no IC_50_ value could be obtained for non-irradiated **6**. As expected from the decaging experiments and *in vitro* assay, incubations with free **1** resulted in a lower IC_50_ (470 nM), than for irradiated **6**. In the live-cell assay, it is seen that the activity of RET can be suppressed to lower values than the positive control (in which no growth factor was added), leading to negative activity readings in Fig. 4. This has been reported for comparable live-cell assays, and is likely due to basal (growth factor-independent) RET activity in the cells^24^. To again exclude distortive effects of light on the cells, incubations without inhibitor were exposed to light. No significant decrease in kinase activity could be observed after up to 15 min irradiation (Fig. S2). The difference in activity between irradiated and non-irradiated inhibitor encouraged us to test the effects of photoactivation of **6** in zebrafish embryos.

### Photocontrolled inhibition of ret in zebrafish embryos

The zebrafish (*Danio rerio*) is a popular model organism for studying developmental biology. Reasons for this include its fast early development, embryonic transparency, and mapped genome. Similar to humans^27^ and mice^28^, zebrafish ret is strongly expressed in primary motoneurons, indicating a role in their development^29^,^30^. However, knock-down of ret in the zebrafish using morpholinos have so far failed to show altered motoneuron phenotypes^31^. This is somewhat surprising considering the fact that *Ret* mutant mice show aberrant motoneuron innervation^32^.

**Figure 6:**
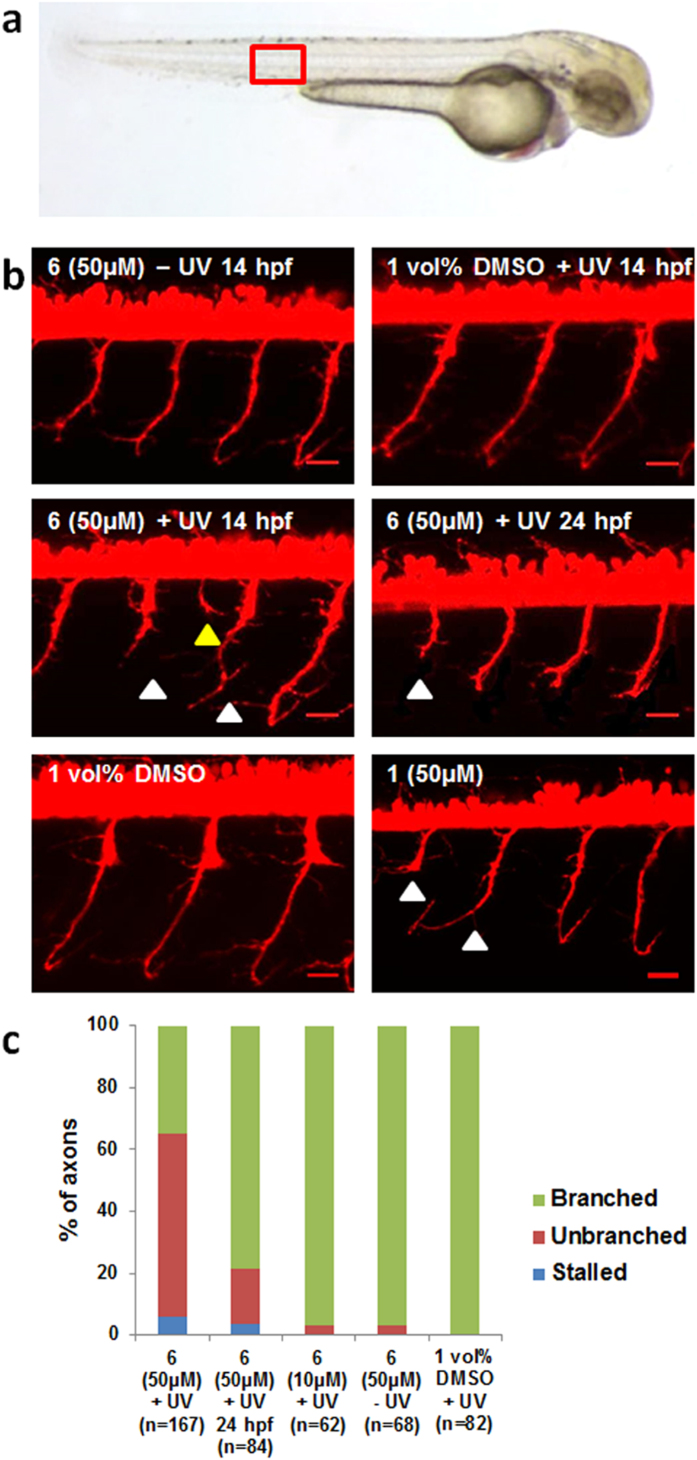
Ret inhibition during development prevents motoneuron extension and axonal pathfinding in the zebrafish. (**a**) Picture showing the area of confocal imaging (red square) (**b**) Confocal image stacks of tg(*olig2:dsRed*) fish showing motoneuron axons after treatment with **6** and **1**. Triangles mark stalling (white) and erroneous (yellow) axons. Scale bars: 20 μm. (**c**) Quantification of axonal phenotypes in the different treatments. n = number of axonal processes quantified.

In the light of this, we chose to investigate the decaging of **6** in the transgenic zebrafish line tg(*olig2:dsRed*)^33^, and any effect this may have on the motoneuron development. The choice of zebrafish line is motivated by the labeling of ventral spinal cord precursor cells that produce motoneurons and oligodendrocytes^34^,^35^, facilitating the assessment of altered motoneuron phenotypes. Transgenic zebrafish embryos were incubated in 10 μM and 50 μM solutions of **6** at 3 hours post fertilization (hpf) for 11 h. We opted for decaging at 14 hpf since primary motoneurons start to sprout growth cones at 18 hpf^36^. We hypothesized that blocking ret signalling at this time point would primarily affect axonal extension and not formation of motoneurons. After washing with fresh medium the embryos were irradiated for 15 min (365 nm). The embryos were left to develop until 2 days post fertilization (dpf) when their spinal cords were scanned with confocal imaging. Embryos incubated with 50 μM **6** and irradiated for 15 min at 14 hpf displayed motoneurons with shortened and malformed axons (Fig. 6b).

This phenotype was also observed when embryos were treated with **1** (10 μM) during development or with **1** (50 μM) for 90 min at 6 hpf (Fig. 6), indicating that the effect of irradiated **6** was a result of released inhibitor. Apart from altered axonal extensions, these embryos developed normally and formed motoneurons, showing that the effect of ret inhibition was specific to motoneuron extension. Non-irradiated embryos exposed to **6** and irradiated embryos in 1 vol% DMSO without compound did not show any phenotypic anomalies and displayed normal motoneuron development. This is true also for the embryos incubated with 10 μM **6** and irradiated for 15 min at 14 hpf (Fig. 6). The quantitative data is shown in Fig. 6c. Altogether, these results clearly show that **6** can be absorbed by the embryo and that incubation with **6** alone (at 50 μM) or irradiation (15 min at 365 nm) alone does not affect motoneuron development of embryos. Furthermore, the concentration of **6** is clearly influential for the development of the irradiated embryos, as 10 μM had virtually no effect when contrasted to 50 μM. We also irradiated embryos at 24 hpf after 10 h of incubation with **6** and subsequent wash (Fig. 6). The olig2-positive cells of these embryos exhibited similar, but less severe, axonal phenotypes to those treated at 14 hpf, indicating that the role of ret on motoneuron extension was reduced at this time point. This highlights the importance of *when* the inhibitor is activated during the development cycle, which adds to the appeal of using a caged approach, allowing for very precise temporal control.

In conclusion, we have developed a caged RET kinase inhibitor that is soluble in aqueous buffer. The compound was shown to inhibit RET *in vitro* with a 12-fold difference between irradiated and non-irradiated compound. A clear difference between inhibition with and without light was also demonstrated in a whole cell assay. Moreover, the compound can be activated with light *in vivo* in zebrafish embryos. The non-irradiated compound does not affect axonal extension at the concentrations used, while decaging results in inhibition of motoneuron development. The time of release was shown to be essential for the inhibition process, highlighting the significance of a photocontrolled approach.

## Methods

### General considerations for the synthesis section

All commercial chemicals were used without prior purification. CH_2_Cl_2_ was distilled from calcium hydride. THF was distilled from sodium/benzophenone. Commercial dry acetonitrile and DMF was used. K_2_CO_3_ was oven-dried before use. Polymer supported base Amberlite IRA-67 (5.6 mmol/g) was purchased from Sigma Aldrich. Reactions were monitored by TLC (Merck silica gel 60 F254) and analyzed under UV (254 nm). Microwave reactions were performed in a Biotage Initiator reactor with fixed hold time. Column chromatography was performed by manual flash chromatography (wet-packed silica, 0.04–0.063 mm) or by automated column chromatography on a Biotage SP-4 instrument using pre-packed silica columns. Analytical high-performance liquid chromatography (HPLC) analysis was carried out on a Waters separation module 2690 connected to a Waters photodiode array detector 996 using an Atlantis® 5 μm C18 AQ (250*4.6 mm) column eluting with a gradient of 20–100% acetonitrile in water using 0.1% TFA as buffer. ^1^H- and ^13^C-NMR spectra were obtained at 400 and 100 MHz respectively, using a Varian 400/54 spectrometer. For compound **6**, additional ^13^C-NMR experiments were acquired on a Bruker Advance III HD 800 MHz spectrometer. All reactions where photolabile protecting groups were involved were carried out avoiding direct light, i.e. covering reaction vessels and columns with aluminum foil and working with the fume hood lamp turned off (ceiling lamp was left on).

### Synthesis of ethyl-2-(4-((((1-isopropyl-3-(phenylethynyl)-1H-pyrazolo[3,4-d]pyrimidin-4-yl)carbamoyl)oxy)methyl)-2-methoxy-5-nitrophenoxy)acetate (5)

Following a procedure for carbamate formation^22^, **1** (51 mg, 0.18 mmol) was dissolved in DMF (1.5 ml) in a MW-vial (0.5–2 ml) and CDI (84 mg, 0.52 mmol) was added. The vial was capped, flushed with nitrogen and heated at 105 °C. After 2 h, the vial was removed from the heat source and a solution of **4** (151 mg, 0.53 mmol) in DMF (1 ml) was added after 5 min. The reaction mixture was stirred at room temperature for 19 h at which point full consumption of **1** was confirmed by TLC (5% methanol in CHCl_3_). The reaction mixture was poured in to ice cold water (40 ml) which resulted in precipitation, ethyl acetate (100 ml) was added, the precipitate was dissolved and the phases were separated. The aqueous phase was extracted with ethyl acetate (50 ml) and the organic phases were pooled and concentrated. The resulting residue was dissolved in CH_2_Cl_2_ and passed through a silica column (10 g) eluting with 2% methanol in CH_2_Cl_2_. After evaporation of the solvents, the resulting yellow residue was washed with ice cold acetonitrile to provide **5** as a white solid (54 mg, 50%). ^1^H NMR (400 MHz, CDCl_3_, δ): 8.77 (s, 1 H), 8.50 (s, 1H), 7.69 (s, 1H), 7.62 (d, *J *= 6.9 Hz, 2H), 7.46 – 7.34 (m, 3H), 7.27 (s, 1H), 5.74 (s, 2H), 5.22 (sept., *J *= 6.6 Hz, 1H), 4.75 (s, 2H), 4.28 (q, *J *= 7.1 Hz, 2H), 3.89 (s, 3H), 1.61 (d, *J *= 6.6 Hz, 6H), 1.31 (t, *J *= 7.1 Hz, 3H); ^13^C NMR (100 MHz, CDCl_3_, δ): 167.9, 156.2, 154.1, 152.9, 152.5, 150.3, 146.9, 140.1, 131.8, 130.0, 129.0, 127.2, 125.1, 121.0, 112.9, 110.9, 104.0, 96.2, 80.5, 66.3, 65.2, 61.9, 56.7, 50.2, 22.1, 14.3; HRMS (*m/z):* [M + H]^+^ calculated for C_29_H_28_N_6_O_8_, 589.2047; found, 589.2052.

### Synthesis of 2-(4-((((1-isopropyl-3-(phenylethynyl)-1H-pyrazolo[3,4-d]pyrimidin-4-yl)carbamoyl)oxy)methyl)-2-methoxy-5-nitrophenoxy)acetic acid (6)

To a suspension of **5** (38 mg, 0.06 mmol) in THF (1 ml) and water (1 ml) was added LiOH monohydrate (6 mg, 0.14 mmol). The reaction mixture was stirred at room temperature for 20 min. Full consumption of **5** was confirmed by TLC (5% methanol in CHCl_3_). The solvents were removed, the residue was taken up in water (10 ml) and the pH was set to 3–5 with HCl (aq., 0.1 M). The aqueous phase was extracted with ethyl acetate (3 × 15 ml) and the organic phases were pooled and concentrated. The white residue was dissolved in CHCl_3_ (60 ml) and MQ-water (10 ml), the aqueous phase was washed with ethyl acetate (100 ml), the organic phases were pooled and the solvents were removed to provide **6** as a white solid (29 mg, 80%) after drying under vacuum overnight. ^1^H NMR (400 MHz, DMSO-*d*_6_, δ): 13.21 (br s, 1H), 10.98 (br s, 1H), 8.78 (s, 1H), 7.64 (s, 1H), 7.56–7.49 (aa’, part of aa’bb’c, 2H), 7.45–7.39 (c, part of aa’bb’c, 1H), 7.38–7.30 (bb’, part of aa’bb’c, 2H), 7.22 (s, 1H), 5.45 (s, 2H), 5.19 (sept., *J *= 6.9 Hz, 1H), 4.84 (s, 2H), 3.84 (s, 3H), 1.53 (d, *J *= 6.6 Hz, 6H); ^13^C NMR (100 MHz, DMSO-*d*_6_, δ): 169.6, 154.9 (broad), 153.5, 153.1 (2C, broad), 152.8 (broad), 146.2, 138.8, 131.2, 129.4, 128.7, 126.9, 126.8, 121.2, 110.8, 109.6, 107.1, 93.0, 81.9, 65.4, 63.9, 56.2, 49.4, 21.7; HRMS (*m/z):* [M + H]^+^ calculated for C_27_H_24_N_6_O_8_, 561.1734; found, 561.1710.

### Spectroscopic details

Steady state absorption measurements (for thermal stability assessment of **6**, see Fig. S3) were carried out on a Cary Bio 50 UV/vis spectrometer equipped with a Varian PCB 1500 Water Peltier System thermostat for temperature control. Sample volume was 3.0 mL. All UV-induced decaging was achieved with a hand-held UVP UV-lamp model UVGL-25, delivering 365 nm light with 1.5 mW/cm^2^ flux *ca.* 1 cm from the lamp. Irradiation in the assays was accomplished by placing the lamp directly on top of the sample plates/dishes. It should be noted that the exact light conditions in the assays may differ slightly depending on the type of plates/dishes used (see Methods sections below for plate specifications).

### Enzyme incubation

Human recombinant RET expressed in Sf9 insect cells (Specific Activity: 220 nmol^−1^min^−1^mg^−1^), substrate (IGFlRtide), and ATP were purchased in an assay ready kit from Promega^37^ (Promega Corporation, Madison WI 53711 USA) and used as received. Incubations were performed in 40 mM Tris buffer (pH 7.4, 1 vol% DMSO) supplemented with 50 μM DTT and 20 mM MgCl_2_ in a white flat-bottom 96-well plate. Incubation volume was 25 μL. Brief procedure; Kinase (0.8 μg/mL), substrate (40 μg/mL), ATP (10 μM) and inhibitor were combined and incubated for 30 min at room temperature in the dark, with shaking. After the kinase reaction, two detection reagents were added in a stepwise manner. The first reagent depleted the remaining (unreacted) ATP in the reaction mix. The second reagent converted the ADP produced in the kinase reaction to ATP and generated a luminescence signal using a luciferase/luciferin reaction. The light generated is proportional to the amount ADP present and, consequently, to the kinase activity. Luminescence was recorded on a BMG Labtech Fluostar Omega luminometer. All incubations were done in duplicates.

### Live-cell incubation

PathHunter express c-RET-GFRα2 functional assay and recombinant human neurturin were purchased from DiscoveRx (DiscoveRx Corporation, Fremont CA 94538 USA) and used according to instructions. For a thorough description of the key concepts of the assay, see reference 24. Incubations were performed in the provided assay buffer (1 vol% DMSO) in a white flat/clear-bottom 96-well plate. Incubation volume was 110 μL. Brief procedure; Cells were thawed and diluted to *ca.* 100 000 cells/mL, followed by plating and acclimatization in 37 °C, 5% CO_2_, humidified, environment for 48 h. Thereafter, the inhibitor was added and the plate was returned to 37 °C for 3 h. The cells were then stimulated with the GDNF-family growth factor neurturin (applied concentration: 15 ng/mL = EC_80_) and incubated 3 h at 22 °C in the dark. An activity-correlating luminescence signal was induced using the detection reagent provided in the kit according to the recommended protocol. Luminescence was recorded on a BMG Labtech Fluostar Omega luminometer. All incubations were done in duplicates.

### Zebrafish treatment and *in vivo* imaging

All experimental procedures were carried out in compliance with relevant guidelines and regulations of the Swedish National Board for Laboratory Animals. The protocols were also approved by the medical ethics committee of University of Gothenburg. Zebrafish (*Danio rerio*) embryos of strain tg(*olig2:dsRed*)^32^ were kept at the Institute of Neuroscience and Physiology, University of Gothenburg. Fertilized eggs were collected and kept in petri dishes containing embryo medium (EM; 5.03 mM NaCl, 0.17 mM KCl, 0.33 mM CaCl_2_·2H_2_O, 0.33 mM MgCl_2_·6H_2_O), in darkness, in an incubator at 28.5 °C. Eggs were incubated in either **6** or 1% DMSO from 3 hours post-fertilization (hpf) and then dechorinated at 9 hpf to allow for better compound uptake. After 11  hours of incubation, 14 hpf embryos were washed three times with EM and moved to clean petri dishes containing 1-phenyl 2-thiourea (PTU, 0,003%, Sigma) and diluted in EM to prevent pigmentation. Embryos were irradiated for 15 min (365 nm) and then kept in the incubator until time of confocal imaging. A second set of animals were incubated in **6** from 14 hpf to 24 hpf, after which animals were washed and illuminated in the same way as the first group. Prior to imaging at 2 days post-fertilization (dpf), the embryos were anesthetized using 0.02% tricaine methanesulfonate (MS-222) diluted in EM and embedded in low-melting agarose. The imaging was performed on a ZEISS LSM710 confocal microscope at the Centre for Cellular Imaging at the Sahlgrenska Academy. Stacks of labeled spinal cord neurons were collected using a 561 nm laser. Maximal intensity pictures of spinal cord hemisegments and axonal phenotypes were analyzed using ImageJ (U. S. National Institutes of Health, Bethesda, Maryland, USA) according to Abramsson *et al.* 2013^38^.

## Additional Information

**How to cite this article**: Bliman, D. *et al.* A Caged Ret Kinase Inhibitor and its Effect on Motoneuron Development in Zebrafish Embryos. *Sci. Rep.* **5**, 13109; doi: 10.1038/srep13109 (2015).

## Supplementary Material

Supplementary Information
