# Supplementary material for: A Caged Ret Kinase Inhibitor and its Effect on Motoneuron Development in Zebrafish Embryos
Source: Sci Rep. 2015 Aug 24;5:13109. doi: 10.1038/srep13109 (PMC4547397; doi:10.1038/srep13109)
Supplement: Supplementary Information [file srep13109-s1.pdf]

# Supporting Information for:

# A Caged Ret Kinase Inhibitor and its Effect on Motoneuron Development in Zebrafish Embryos

David Bliman[a], Jesper R. Nilsson[b], Petronella Kettunen[c], Joakim Andréasson[b]* and Morten Grøtli[a]*

[a] Department of Chemistry and Molecular Biology, University of Gothenburg, SE-412 96 Gothenburg, Sweden

[b] Department of Chemistry and Chemical Engineering, Physical Chemistry, Chalmers University of Technology, SE-412 96 Gothenburg, Sweden

[c] Institute of Neuroscience and Physiology, Sahlgrenska Academy at University of Gothenburg, SE-413 45 Gothenburg, Sweden

*Correspondence to a-son@chalmers.se and grotli@chem.gu.se

Content

Entry ... Page

1 Synthesis ... S2

2 Fitting of dose-response data ... S7

3 Molecular modeling ... S7

4 Ethical statement ... S7

5 Supplementary figures ... S8

6 $^{1}$H- and $^{13}$C-spectra of previously unpublished compounds ... S11

7 References ... S21

## 1. Synthesis

### General

All commercial chemicals were used without prior purification. $CH_2Cl_2$ was distilled from calcium hydride. THF was distilled from sodium/benzophenone. Commercial dry acetonitrile and DMF was used. $K_2CO_3$ was oven-dried before use. Polymer supported base Amberlite IRA-67 (5.6 mmol/g) was purchased from Sigma Aldrich. Reactions were monitored by TLC (Merck silica gel 60 F254) and analyzed under UV (254 nm). Microwave reactions were performed in a Biotage Initiator reactor with fixed hold time. Column chromatography was performed by manual flash chromatography (wet-packed silica, 0.04–0.063 mm) or by automated column chromatography on a Biotage SP-4 instrument using pre-packed silica columns. Analytical high-performance liquid chromatography (HPLC) analysis was carried out on a Waters separation module 2690 connected to a Waters photodiode array detector 996 using an Atlantis® 5 µm C18 AQ (250*4.6 mm) column eluting with a gradient of 20-100% acetonitrile in water using 0.1% TFA as buffer. $^{1}$H- and $^{13}$C-NMR spectra were obtained at 400 and 100 MHz respectively, using a Varian 400/54 spectrometer. For compound **6**, additional $^{13}$C-NMR experiments were acquired on a Bruker Advance III HD 800 MHz spectrometer. All reactions where photolabile protecting groups were involved were carried out avoiding direct light, i.e. covering reaction vessels and columns with aluminium foil and working with the fume hood lamp turned off (ceiling lamp was left on).

### 3-iodo-1H-pyrazolo[3,4-d]pyrimidin-4-amine

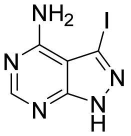

1H-pyrazolo[3,4-d]pyrimidin-4-amine (6.70 g, 49.6 mmol) and *N*-iodosuccinimide (12.4 g, 55.0 mmol) were dissolved in dry DMF (140 ml). The solution was heated to 80 °C for 5 h and 30 min and the organic solvent was then evaporated and co-distilled with toluene several times. Ethanol (99%) was added to the solution and the product was filtered. The solid material was rinsed several times with ethanol (99%) and was then dried under vacuum to give 3-iodo-1H-pyrazolo[3,4-d]pyrimidin-4-amine as a pale yellow solid (8.21 g, 63%). Spectroscopic data was in accordance with the literature.[1]

### 3-iodo-1-isopropyl-1H-pyrazolo[3,4-d]pyrimidin-4-amine

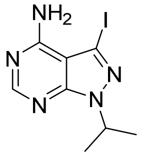

Following a previously published procedure[2], 2-chloropropane (0.39 ml, 4.27 mmol) was added to a suspension of 3-iodo-1H-pyrazolo[3,4-d]pyrimidin-4-amine (1.00 g, 3.83 mmol) and $K_2CO_3$ (1.06 g, 7.67 mmol) in DMF (10 ml). The reaction was heated at 200 °C for 5 min in a microwave reactor. The reaction mixture was then allowed to cool to room temperature and additional 2-chloropropane (0.18 ml, 1.97 mmol) was added. The reaction was heated again at 200 °C for 5 min. The reaction mixture was then diluted with DMF, filtered and the solvents were removed at reduced pressure. Purification by flash column chromatography (0-5% methanol in $CHCl_3$) gave 3-iodo-1-isopropyl-1H-pyrazolo[3,4-d]pyrimidin-4-amine as a yellow solid (849 mg, 74%). Spectroscopic data was in accordance with the literature[2].

### 1-isopropyl-3-(phenylethynyl)-1H-pyrazolo[3,4-d]pyrimidin-4-amine (1)

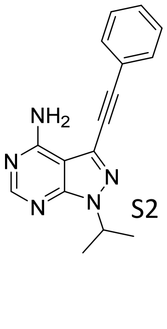

Following a previously published procedure with modifications[3], 3-iodo-1-isopropyl-1H-pyrazolo[3,4-d]pyrimidin-4-amine (201 mg, 0.66 mmol), $Pd(PPh_3)_4$ (37 mg, 0.03 mmol) and CuI (12 mg, 0.06 mmol) was dissolved in freshly distilled THF (10 ml), Amberlite IRA-67 (478 mg, 2.68 mmol) was added, the vial was capped and nitrogen

was bubbled through the reaction mixture. Phenylacetylene (0.22 ml, 2.00 mmol) was added with syringe and the reaction mixture was heated at 60 °C for 18 h at which point full conversion was confirmed by TLC (10 % methanol in $CHCl_3$). The reaction mixture was filtered, the solid phase washed with 10% methanol in $CHCl_3$ and the solvents were removed by rotary evaporation. Purification by flash column chromatography (0-5% methanol in $CHCl_3$) afforded **1** as an off white solid (165 mg, 90%). $^1$H NMR (400 MHz, $CDCl_3$ δ): 8.35 (s, 1H), 7.63-7.54 (m, 2H), 7.44-7.34 (m, 3H), 6.15 (br s, 1H), 5.15 (sept., *J*=6.7 Hz, 1H), 1.58 (d, *J*=6.7 Hz, 6H); $^{13}$C NMR (100 MHz, $CDCl_3$, δ): 157.9, 156.2, 152.6, 131.9, 129.6, 128.7, 126.1, 121.8, 102.1, 94.0, 81.1, 49.6, 22.1.

**4,5-dimethoxy-2-nitrobenzyl-(1-isopropyl-3-(phenylethynyl)-1H-pyrazolo[3,4-d]pyrimidin-4-yl)carbamate (2)**

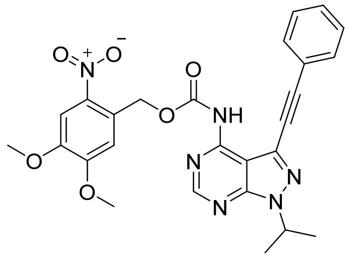


NVOC-Cl (88 mg, 0.32 mmol) was added to a solution of tetrazole (0.85 ml solution in acetonitrile, 0.45 M) and $Et_3N$ (51 µl, 0. 37 mmol) in THF (4 ml) under nitrogen at 0 °C[4]. The ice bath was removed after 10 min and stirred for an additional 40 min at room temperature. The white precipitate that formed was filtered off through celite, washed with THF and the resulting solution was concentrated to 8 ml. This solution was added to a solution of **1** (68 mg, 0.25 mmol) in THF (3 ml) under nitrogen. The reaction mixture was heated at 70 ºC for 48 h, and then stirred at room temperature overnight. The reaction was not complete as observed by LCMS and more NVOC-tetrazolide synthesized as above with NVOC-Cl (135 mg, 0.49 mmol), tetrazole (1.1 ml solution in acetonitrile, 0.45 M) and $Et_3N$ (68 µl, 0.49 mmol) in THF (5 ml) was added to the reaction mixture. The reaction was refluxed for 5 h 15 min and then stirred at room temperature overnight. The white solid which formed was filtered off and the solvents were removed to give a yellow residue. After two consecutive silica columns (1% methanol and 1% toluene in $CH_2Cl_2$, then 0-5 % methanol in $CHCl_3$), the remaining impurities were removed by suspending the crude in acetonitrile and isolating **2** by filtration as an off white solid (53 mg, 42 %). $^1$H NMR (400 MHz, $CDCl_3$, δ): 8.76 (s, 1H), 8.50 (br s, 1H), 7.74 (s, 1H), 7.65-7.59 (m, 2H), 7.45-7.34 (m, 3H), 7.23 (s, 1H), 5.74 (s, 2H), 5.22 (sept., *J*=6.7 Hz, 1H), 3.97 (s, 3H), 3.89 (s, 3H), 1.61 (d, *J*=6.7 Hz); $^{13}$C NMR (100 MHz, $CDCl_3$, δ): 156.0, 153.6, 152.8, 152.5, 150.4, 148.8, 140.4, 131.8, 129.8, 128.9, 125.9, 125.2, 121.0, 112.2, 108.4, 104.0, 96.1, 80.5, 65.2, 56.6 (2C), 50.2, 22.1; (note that the methoxy carbons (56.6 ppm) overlaps in $^{13}$C as confirmed by HSQC). HRMS (*m/z)*: $[M + H]^+$ calculated for $C_{26}H_{24}N_6O_6$, 517.1835; found, 517.1801.

**1-(2-((tert-butyldimethylsilyl)oxy)ethyl)-3-iodo-1H-pyrazolo[3,4-d]pyrimidin-4-amine**

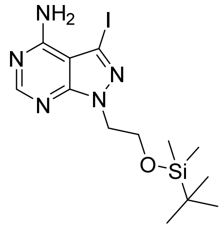


DMF (2 ml) and (2-Bromoethoxy)-tert-butyldimethylsilane (77 µl, 0.46 mmol) were added to a capped microwave vial containing 3-iodo-1H-pyrazolo[3,4-d]pyrimidin-4-amine (101 mg, 0.39 mmol) and $Cs_2CO_3$ (154 mg, 0.47 mmol) under nitrogen. The reaction mixture was stirred at room temperature for 48 h. The solvents were removed, water (40 ml) and $CHCl_3$ (70 ml) were added to the brown residue and the biphasic mixture was stirred vigorously to dissolve the material. The phases were separated and the aqueous phase was extracted with $CHCl_3$. The organic phases were pooled, washed with brine and dried over $Na_2SO_4$. Removal of the solvents by rotary evaporation followed by manual flash column chromatography (5% methanol in $CHCl_3$) provided 1-(2-((tert-butyldimethylsilyl)oxy)ethyl)-3-iodo-1H-pyrazolo[3,4-d]pyrimidin-4-amine as an off white solid (121 mg, 74 %). $^1$H NMR (400 MHz, $CDCl_3$, δ): 8.33 (s, 1H), 6.00 (br s, 2H), 4.49 (t, *J* = 5.8 Hz, 2H), 4.02 (t, *J* =5.8 Hz, 2H), 0.75 (s, 9H), -0.11 (s, 6H); $^{13}$C NMR (100 MHz, $CDCl_3$, δ):

157.4, 156.2, 154.7, 104.1, 86.1, 61.5, 49.9, 25.8, 18.2, -5.5; HRMS (*m/z):* $[M + H]^+$ calculated for $C_{13}H_{22}IN_5OSi$, 420.0717; found, 420.0729.

**1-(2-((*tert*-butyldimethylsilyl)oxy)ethyl)-3-(phenylethynyl)-1H-pyrazolo[3,4-d]pyrimidin-4-amine**

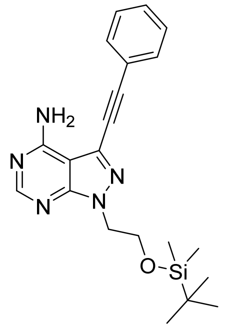


1-(2-((tert-butyldimethylsilyl)oxy)ethyl)-3-iodo-1H-pyrazolo[3,4-d]pyrimidin-4-amine (70 mg, 0.17 mmol), $Pd(PPh_3)_4$ (4 mg, 4 μmol) and CuI (5 mg, 0.03 mmol) were dissolved in freshly distilled THF (2 ml) in a microwave vial. Amberlite IRA-67 (119 mg, 0.666 mmol) was added, the vial was capped and nitrogen was bubbled through the reaction mixture. Phenylacetylene (55 μl, 0.50 mmol) was added by syringe and the reaction mixture was heated at 60 °C for 4 h at which point full consumption of starting material was confirmed by TLC (5% methanol in $CHCl_3$). The reaction mixture was filtered through a silica plug which was washed with 10% methanol in $CHCl_3$ and the solvents were removed by rotary evaporation. Purification by manual flash column chromatography (2% methanol in $CHCl_3$) provided 1-(2-((*tert*-butyldimethylsilyl)oxy)ethyl)-3-(phenylethynyl)-1H-pyrazolo[3,4-d]pyrimidin-4-amine as an off white solid (63 mg, 95%). $^1H$ NMR (400 MHz, $CDCl_3$, δ): 8.37 (br s, 1H), 7.63-7.56 (m, 2H), 7.46-7.37 (m, 3H), 6.00 (br s, 2H), 4.53 (t, $J$ = 5.9 Hz, 2H), 4.07 (t, $J$ = 5.9 Hz, 2H), 0.77 (s, 9H), -0.08 (s, 6H); $^{13}C$ NMR (100 MHz, $CDCl_3$, δ): 158.0, 156.4, 154.1, 131.9, 129.6, 128.7, 126.6, 121.7, 101.7, 94.1, 80.9, 61.5, 49.8, 25.8, 18.2, -5.5; HRMS (*m/z):* $[M + H]^+$ calculated for $C_{22}H_{27}N_5OSi$, 394.2063; found, 394.2076.

**4,5-dimethoxy-2-nitrobenzyl-(1-(2-((*tert*-butyldimethylsilyl)oxy)ethyl)-3-(phenylethynyl)-1H-pyrazolo[3,4-d]pyrimidin-4-yl)carbamate**

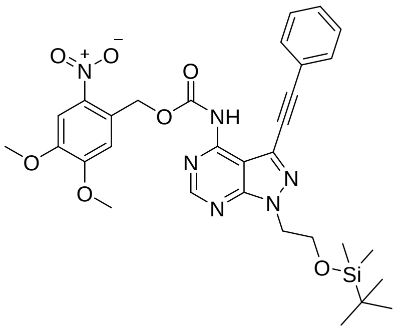


NVOC-Cl (152 mg, 0.55 mmol) was added to a solution of tetrazole (1.1 ml solution in AcCN, 0.45M) and $Et_3N$ (75 μl, 0.54 mmol) in THF (7 ml) under nitrogen at 0 °C[4]. The ice bath was removed after 10 min and the reaction mixture was stirred for 60 min at room temperature. The white precipitate which formed was filtered off through Celite, washed with THF and concentrated (to approx. 8 ml). The solution was added to a solution of 1-(2-((tert-butyldimethylsilyl)oxy)ethyl)-3-(phenylethynyl)-1H-pyrazolo[3,4-d]pyrimidin-4-amine (100 mg, 0.25 mmol) in THF (5 ml) under nitrogen. The reaction mixture was heated at 70 °C for 4 h. The solvents were removed to give a yellow residue which was redissolved in ethyl acetate (50 ml), washed with sat. $NaHCO_3$ (aq., 40 ml), brine (40 ml), dried over $Na_2SO_4$. The solvents were removed by rotary evaporation and purification by manual column chromatography (1-10% ethyl acetate in $CH_2Cl_2$) provided 4,5-dimethoxy-2-nitrobenzyl-(1-(2-((*tert*-butyldimethylsilyl)oxy)ethyl)-3-(phenylethynyl)-1H-pyrazolo[3,4-d]pyrimidin-4-yl)carbamate as a pale yellow solid (105 mg, 65 %). $^1H$ NMR (400 MHz, $CDCl_3$, δ): 8.76 (s, 1H), 8.49 (br s, 1H), 7.72 (s, 1H), 7.64-7.55 (m, 2H), 7.47-7.31 (m, 3H), 7.22 (s, 1H), 5.73 (s, 2H), 4.57 (t, $J$ = 5.6 Hz, 2H), 4.08 (t, $J$ = 5.6 Hz, 2H), 3.95 (s, 3H), 3.88 (s, 3H), 0.72 (s, 9H), -0.13 (s, 6H); $^{13}C$ NMR (100 MHz, $CDCl_3$, δ): 156.5, 154.4, 153.6, 152.5, 150.3, 148.8, 140.5, 131.8, 129.9, 128.9, 125.8, 120.9, 112.4, 108.4, 103.6, 96.3, 80.2, 65.3, 61.3, 56.58, 56.56, 50.1, 25.7, 18.1, -5.5; (note that the methoxy carbons overlaps in $^{13}C$ as confirmed by HSQC). HRMS (*m/z):* $[M + H]^+$ calculated for $C_{31}H_{36}N_6O_7Si$, 633.2493; found, 633.2524.

**4,5-dimethoxy-2-nitrobenzyl-(1-(2-hydroxyethyl)-3-(phenylethynyl)-1H-pyrazolo[3,4-d]pyrimidin-4-yl)carbamate (3)**

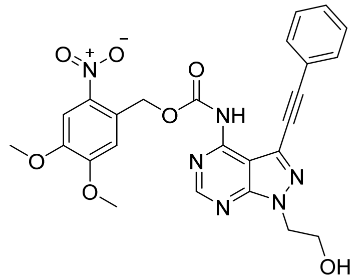


TBAF in THF (190 μl, 1M, 0.19 mmol) was added to a solution of (1-(2-((tert-butyldimethylsilyl)oxy)ethyl)-3-(phenylethynyl)-1H-pyrazolo[3,4-d]pyrimidin-4-yl)carbamate (60 mg, 0.09 mmol) in dry THF (2 ml). The reaction mixture was stirred at room temperature for 3 h. Full consumption of starting material was confirmed by TLC (10% methanol in $CHCl_3$). The solvents were removed by rotary evaporation and purification by manual flash column chromatography (10-50% ethyl acetate in $CH_2Cl_2$) provided **3** as an off white solid (17 mg, 35%). $^1$H NMR (400 MHz, DMSO-$d_6$, δ): 10.97 (s, 1H), 8.79 (s, 1H), 7.68 (s, 1H), 7.55-7.48 (aa', part of aa'bb'c, 2H), 7.45-7.39 (c, part of aa'bb'c, 1H), 7.37-7.31 (bb', part of aa'bb'c, 2H), 7.17 (s, 1H), 5.46 (s, 2H), 4.93 (br s, 1H), 4.49 (t, $J$ = 5.6 Hz, 2H), 3.88 (t, $J$ = 6.2 Hz, 2H), 3.92-3.85 (m, 5H), 3.80 (s, 3H); $^{13}$C NMR (100 MHz, DMSO-$d_6$, δ): 155.4, 154.4, 153.4, 153.1, 152.7, 147.8, 139.1, 131.2, 129.5, 128.8, 126.9, 126.4, 121.2, 110.2, 108.1, 107.1, 93.0, 81.9, 64.0, 59.1, 56.2, 56.1, 50.0; HRMS (*m/z):* $[M + H]^+$ calculated for $C_{25}H_{22}N_6O_7$, 519.1623; found, 519.1600.

**4-ethyloxycarbonylmethoxy-5-methoxy-2-nitro-benzylalcohol (4)**

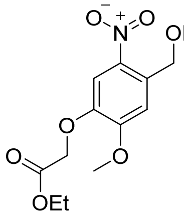


Vanillin (3.00 g, 19.7 mmol) was dissolved in dry acetonitrile (50 ml) in an oven dried two necked 100 ml flask under nitrogen. $K_2CO_3$ (6.54 g, 47.3 mmol) and KI (655 mg, 3.95 mmol) were added and one of the septa was replaced with a condenser. Ethyl bromoacetate (2.6 ml, 23.4 mmol) was added and the mixture was refluxed under nitrogen for 18 h. Full consumption of vanillin was confirmed by TLC (developing the same plate with 33% ethyl acetate in pentane and then 2% methanol in $CHCl_3$). The precipitate that had formed was filtered off and washed with ethyl acetate (3 x 20 ml). The solvents were removed by rotary evaporation, the oily residue was dissolved in diethyl ether (140 ml) and water (40 ml), the phases were separated and the aqueous phase was extracted with diethyl ether (2x 100 ml). The pooled organic phases were washed with water (40 ml), dried over $MgSO_4$ and the solvents were removed by rotary evaporation to yield a yellow thick oil (3.3 g) which was further dried on high vacuum. The product was identified by $^1$H-NMR ($CDCl_3$) and carried to the next step without further purification. Following a literature procedure[5], a mixture of fuming $HNO_3$ (8 ml) and glacial acetic acid (32 ml) was added drop wise with addition funnel at 0 °C (ice bath) to the yellow oil (3.3 g) obtained in the previous step. The ice bath was removed and the yellow reaction mixture was stirred at room temperature for 19 h. Full consumption of starting material was confirmed by TLC (33% ethyl acetate in pentane). The reaction mixture was poured on to crushed ice (approx. 50 ml), diethyl ether (200 ml) was added followed by water (50 ml), the phases were separated and the aqueous phase was extracted with diethyl ether (2x150 ml). The organic phases were pooled and washed with 50 ml portions of sat. $NaHCO_3$ solution (aq.) until bubbling seizes (*Caution! substantial gas evolution*). The pooled organic phases were diluted with diethyl ether (50 ml), washed with brine (50 ml) and dried over $MgSO_4$. Removal of the solvents by rotary evaporation gave a sticky yellow solid which was dissolved in $CHCl_3$. Removal of the solvents gave the crude product as a yellow solid (2.50 g) which was carried to the next step without further purifications. $NaBH_4$ (290 mg, 7.66 mmol) was added to a solution of the crude aldehyde (2.23 g) in THF (37 ml) and absolute EtOH (99.7%, 30 ml) at 0 ºC (ice bath). The reaction mixture was stirred at 0 °C. Full consumption of starting material was confirmed by TLC (50% ethyl acetate in pentane) after 10 min and the reaction was quenched with sat. $NH_4Cl$ (aq., 3 ml) after 15 min at 0 ºC. The mixture was then allowed to reach room temperature and ethanol and THF was removed by rotary evaporation. Water (50 ml) and ethyl acetate (100 ml) were added, the phases were separated and the aqueous phase was extracted with ethyl acetate (2 x 100 ml). The organic phases were pooled, washed with brine (50 ml), dried over $MgSO_4$ and the solvents were removed by rotary evaporation. Purification by manual flash column

chromatography (the crude was dissolved in $CH_2Cl_2$ and loaded from celite, 33-50% ethyl acetate in pentane) provided **4** as a yellow solid (807 mg, 14 % over 3 steps). $^1$H NMR (400 MHz, $CDCl_3$, δ): 7.66 (s, 1H), 7.23 (s, 1H), 4.97 (d, *J* 6.3 Hz, 2H), 4.74 (s, 2H), 4.28 (q, *J* = 7.1 Hz, 2H), 4.01 (s, 3H), 2.60 (t, *J* = 6.4 Hz, 1H), 1.31 (t, *J* = 7.1 Hz, 3H); $^{13}$C NMR (100 MHz, $CDCl_3$, δ): 168.1, 154.5, 146.0, 139.4, 133.9, 111.5, 110.8, 66.4, 62.7, 61.8, 56.6, 14.3; HRMS (*m/z):* $[M + NH_4]^+$ calculated for $C_{12}H_{15}NO_7$, 303.1192; found, 303.1164.

## 2. Fitting of dose-response data

Positive and negative control experiments with and without light exposure (15 min, 365 nm) were run in parallel to the inhibitor incubations. The positive control was identical to the inhibitor experiment but with buffer additions instead of ATP and Neurturin for the cell-free and live-cell incubation, respectively. The negative control was identical to the inhibitor experiment but with inhibitor diluent (DMSO) addition instead of inhibitor. The intensity reading from the positive control was subtracted from the corresponding value from the inhibitor incubation. This was followed by normalization to the negative control reading. The resulting luminescence intensities (*I*) at the applied inhibitor concentrations (*C*) were fitted to the Hill-equation:

$$I = A_1 + \frac{A_2 - A_1}{1 + 10^{(\log C_0 - C)\times p}}$$

A global, error-weighted (instrumental) fit was performed by making the top- ($A_2$) and bottom ($A_1$) asymptotes shared parameters for the included data sets.

## 3. Molecular modeling

Crystal coordinates (PDB:2IVV) were downloaded from the protein data bank (PDB) and prepared using the structure preparation tool in MOE[6] with protonate3D. Compound **1** was docked using the rigid receptor protocol defining the receptor site around the cocrystallized ligand. The default settings (Trianglematcher, rescoring with London dG and refinement with GBVI/WSA dG) were used and the lowest energy pose was selected. The results corresponded well with previously published docking studies[3] using Glide in the Schrödinger suite. Compound **6** was manually superposed over **1** using the phenylethynyl and pyrazolopyrimidine bicyclic system as template.

## 4. Ethical statement

The performed zebrafish experiments were approved by the local Ethics Committee for Animal Experiments in Gothenburg (Göteborgs djurförsöksetiska nämnd), reference number 140/2013, and followed the guidelines of the Swedish National Board for Laboratory Animals.

## 5. Supplementary figures

### 5.1 Decaging of 6

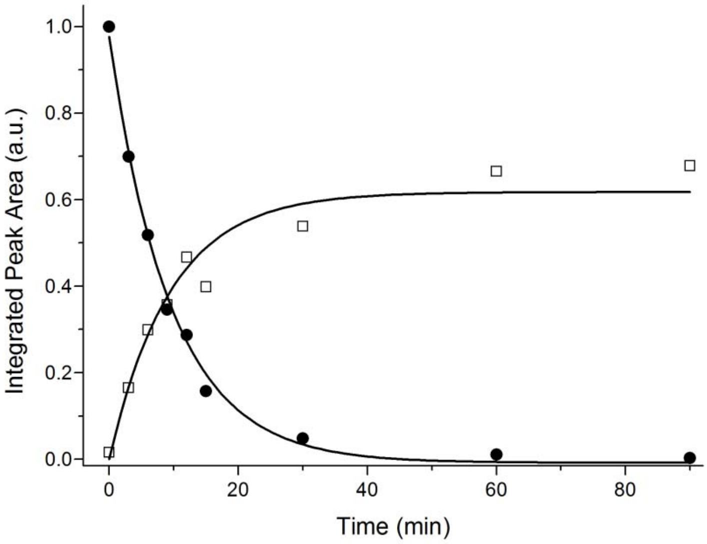


**Figure S1**. Light-induced decaging of **6**. A Tris buffer solution (1vol% DMSO) containing 100 µM **6** was successively subjected to $t$ = 0, 3, 6, 9, 12, 15, 30, 60, and 90 min of 365 nm light. After each irradiation period, an aliquot was drawn and analyzed using HPLC with UV detection. The integrated areas for the chromatogram peaks associated with **1** (hollow squares) and **6** (solid circles) were fitted globally (shared time constant) to a first order exponential (solid line), yielding a time constant of $\tau$ = 9.6 min for the decaging of **6**. It should be noted that the individually extracted time constants for the liberation of **1** and consumption of **6** ($\tau$ = 13.1 and 8.7 min, respectively) do not differ significantly from the globally fitted value.

## 5.2 UV tolerance of RET Assays

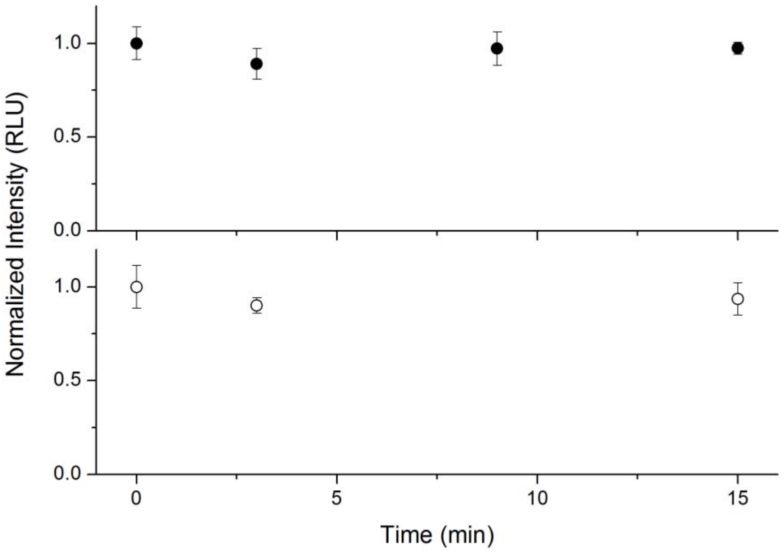


**Figure S2**. UV tolerance of RET incubation assays. Top panel: Cell-free assay; A reaction mixture comprising RET kinase (0.8 µg/mL) and substrate (40 µg/mL) albeit without inhibitor was subjected to $t = 0$, 3, 9, or 15 min 365 nm light. Thereafter, ATP was added (50 µM) and the RET kinase activity was assessed (solid circles). Bottom panel: Live-cell assay; Thawed and acclimatized cells (100 000 cells/mL) without inhibitor was subjected to $t = 0$, 3, or 15 min 365 nm light. Thereafter, Neurturin was added (at $EC_{80}$, determined to 15 ng/mL) and the RET kinase activity was assessed (hollow circles). Error bars are mean ± standard deviation of duplicate samples. It is clear that the applied UV-light has no apparent effect on the enzymatic activity in the cell-free or live-cell assay.

### 5.3 Thermal stability of 6 in 10 mM phosphate buffer, pH 7.

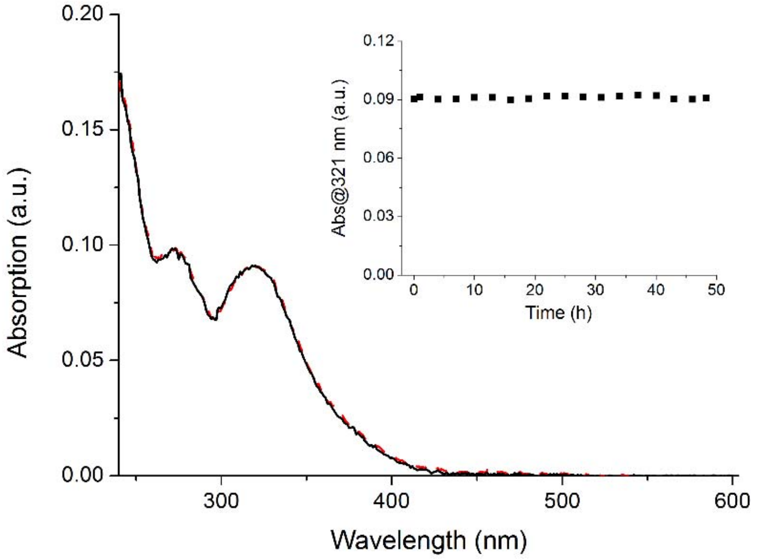


**Figure S3**. UV/vis absorption spectra of **6** in 10 mM phosphate buffer (pH 7.0, 1 vol% DMSO) at $t = 0$ (solid black line), and after $t = 48$ h in 37 ºC (dashed red line). Inset: Absorption at $\lambda = 321$ nm as a function of time. No signs of degradation of **6** were detected under the specified conditions.

## 6. $^{1}$H and $^{13}$C spectra of previously unpublished compounds

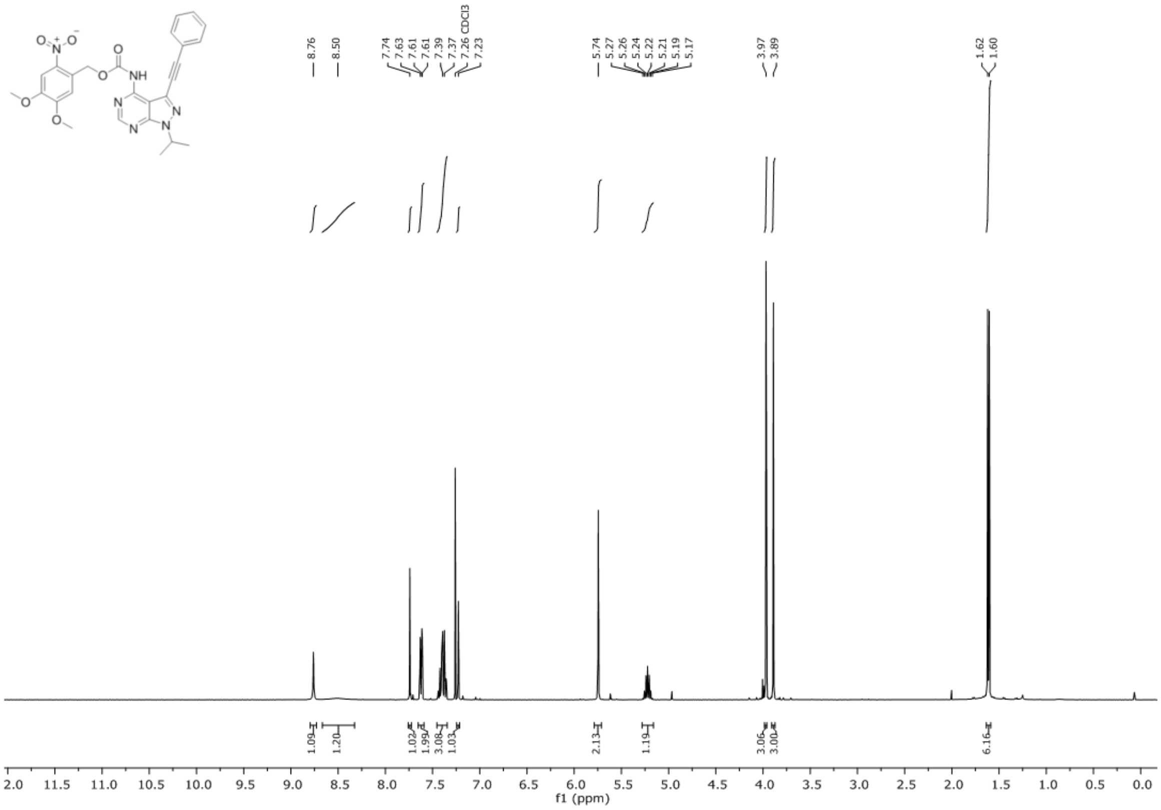


**Figure S4.** $^{1}$H-NMR (400 MHz, $CDCl_3$) spectra of **2**.

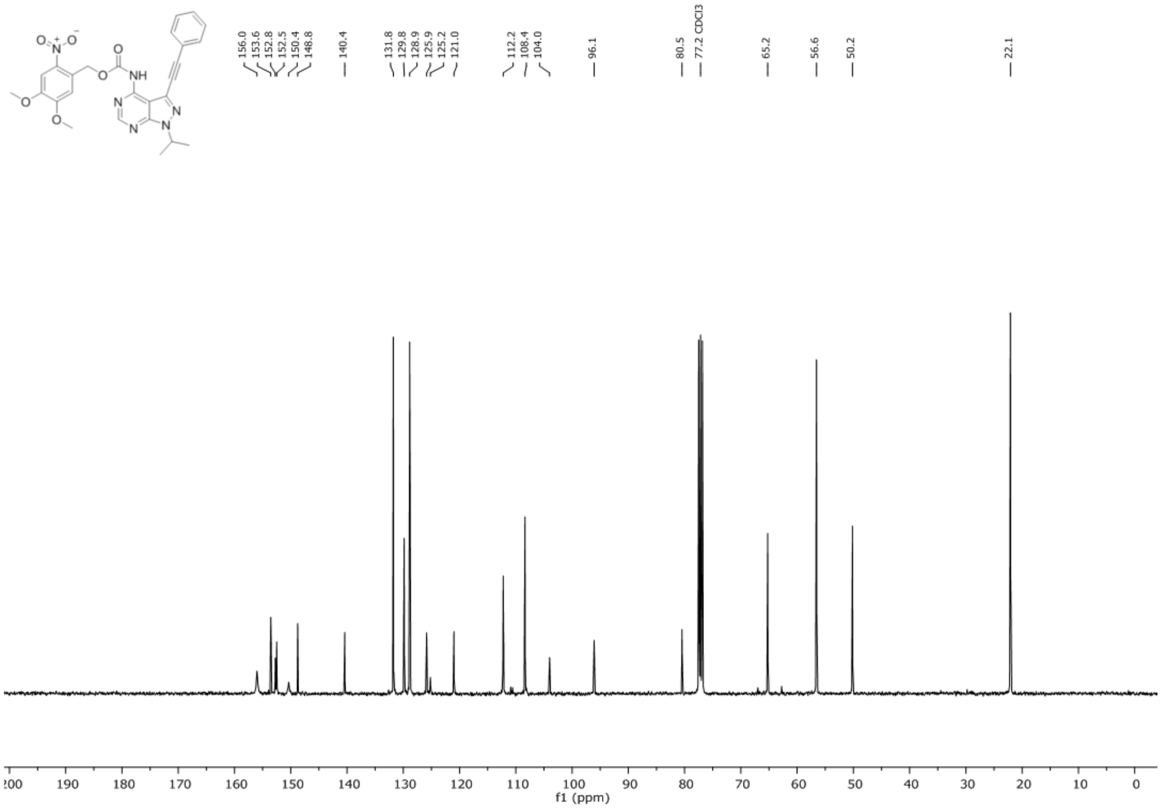


**Figure S5.** $^{13}$C-NMR (100 MHz, $CDCl_3$) spectra of **2**.

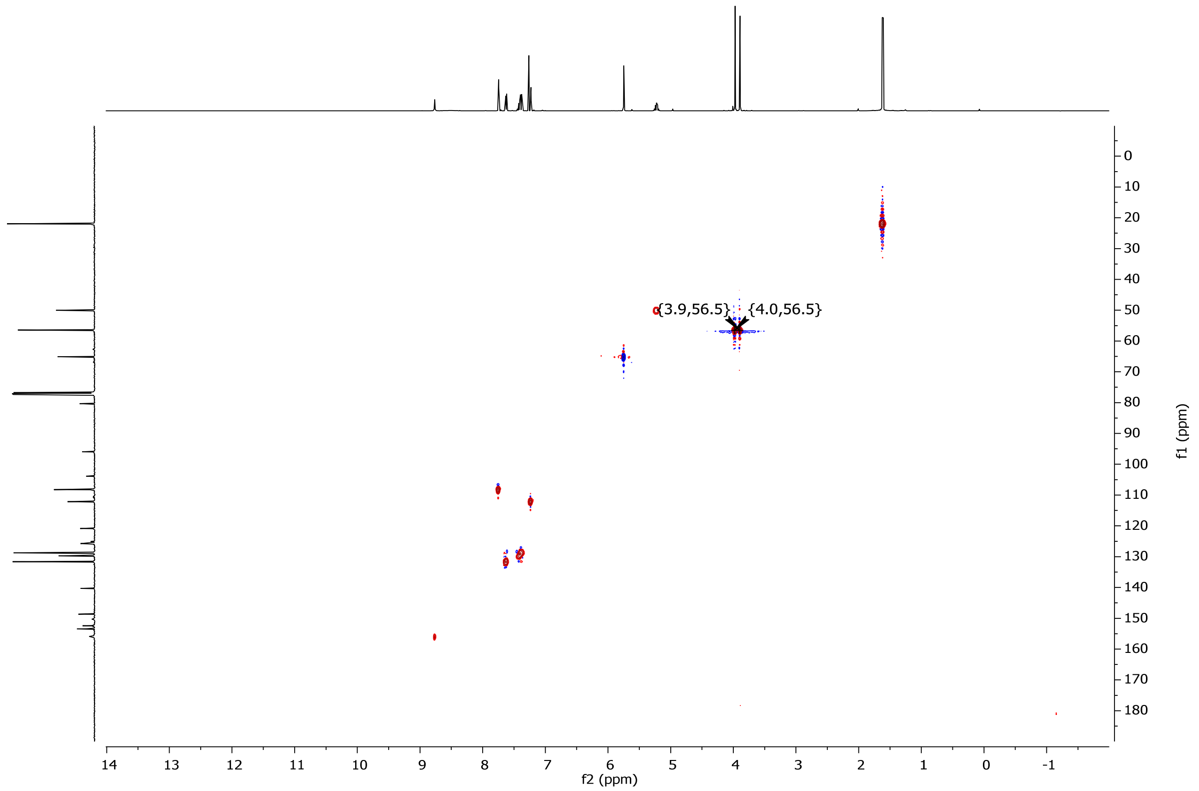


**Figure S6.** gHSQC of **2** confirming the overlap of the methoxy carbon signals.

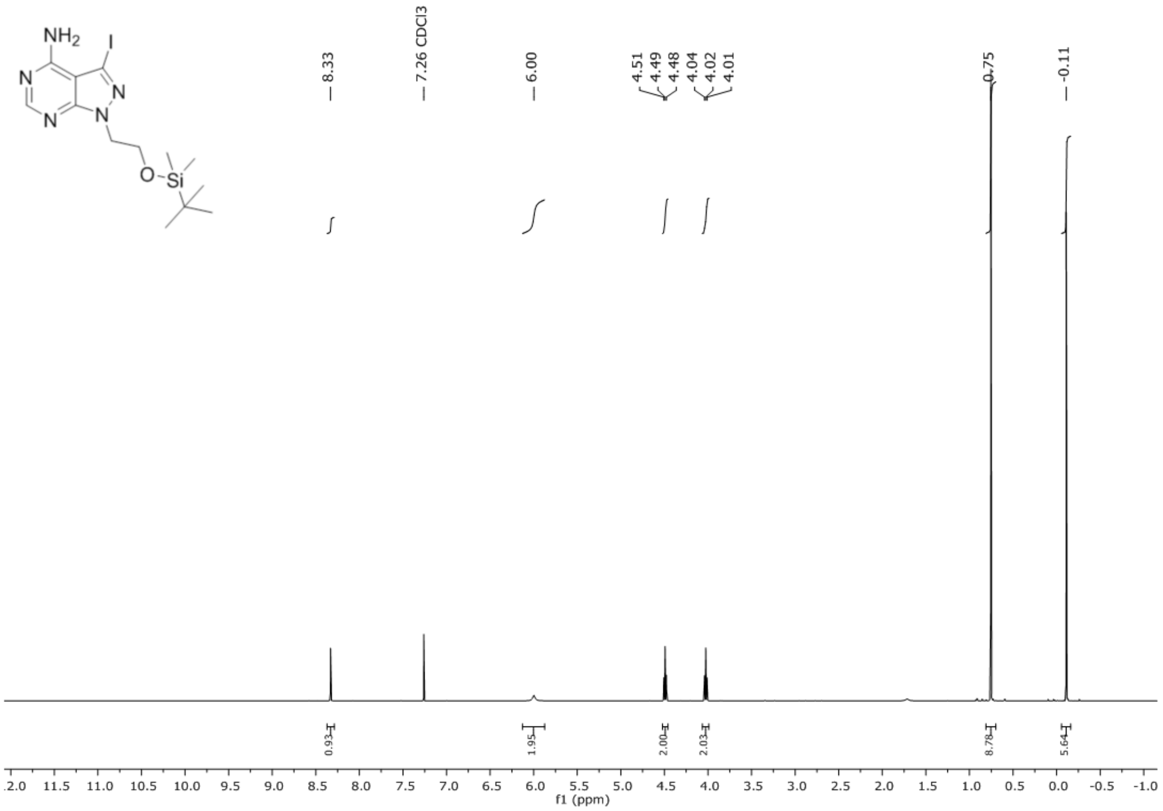


**Figure S7.** $^{1}$H-NMR (400 MHz, $CDCl_3$) spectra of 1-(2-((tert-butyldimethylsilyl)oxy)ethyl)-3-iodo-1H-pyrazolo[3,4-d]pyrimidin-4-amine.

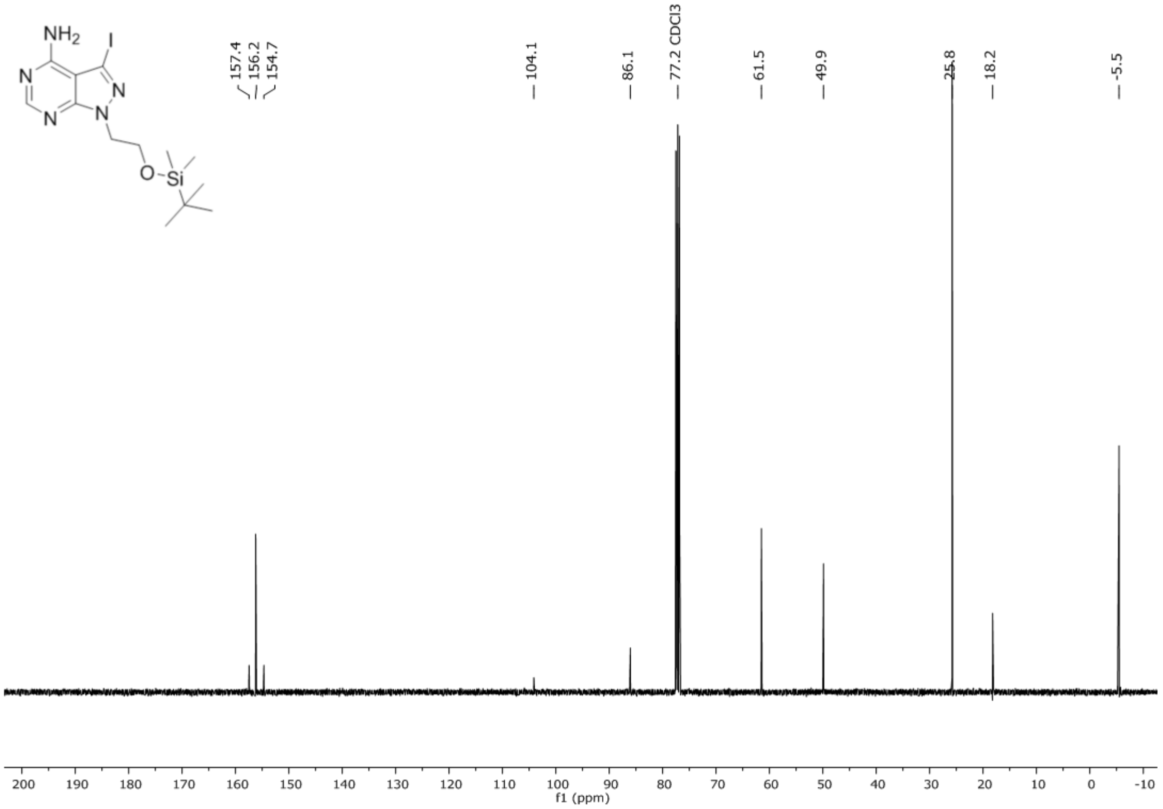


**Figure S8.** $^{13}C$-NMR (100 MHz, $CDCl_3$) spectra of 1-(2-((tert-butyldimethylsilyl)oxy)ethyl)-3-iodo-1H-pyrazolo[3,4-d]pyrimidin-4-amine.

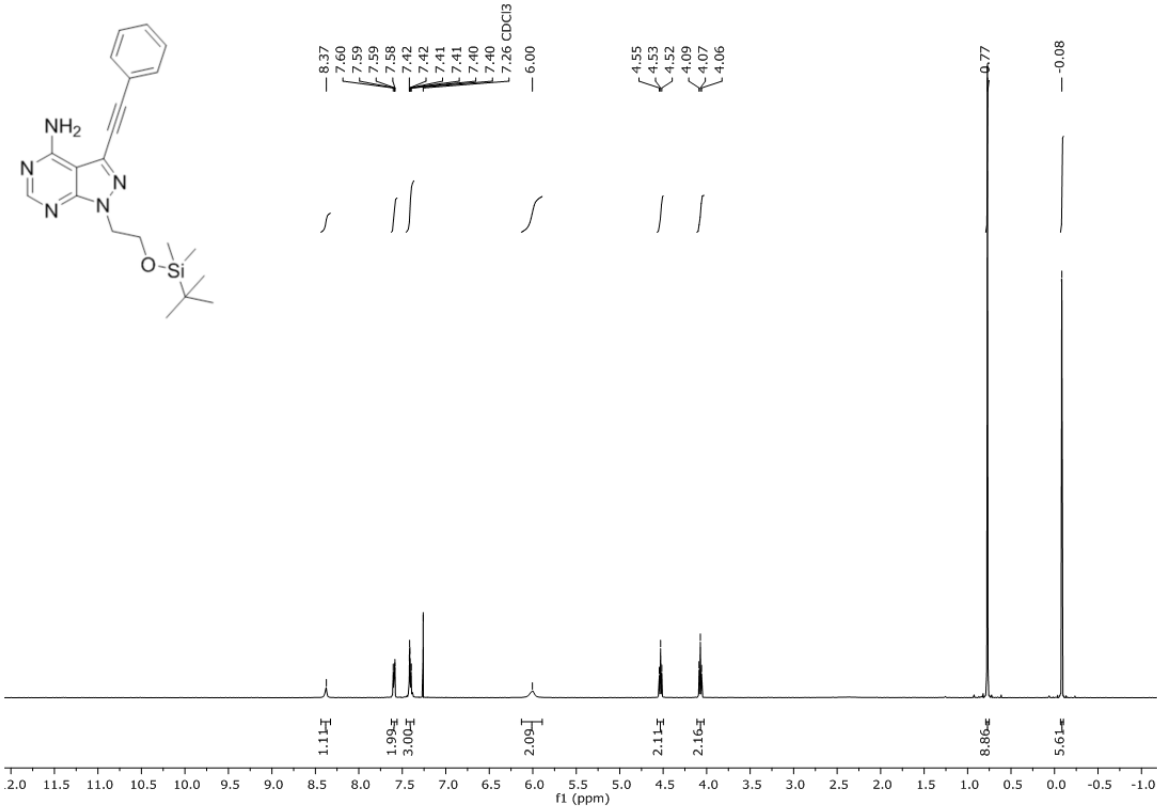


**Figure S9.** $^{1}H$-NMR (400 MHz, $CDCl_3$) spectra of 1-(2-((tert-butyldimethylsilyl)oxy)ethyl)-3-(phenylethynyl)-1H-pyrazolo[3,4-d]pyrimidin-4-amine.

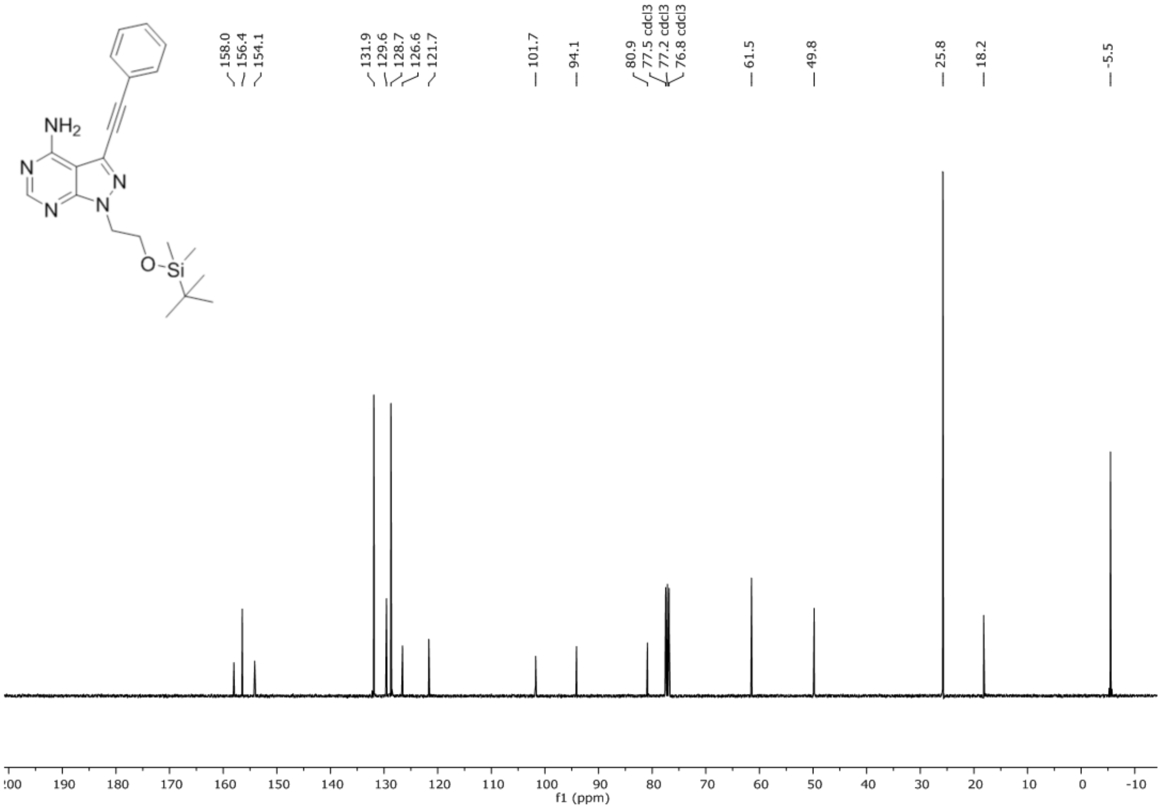


**Figure S10.** $^{13}$C-NMR (100 MHz, $CDCl_3$) spectra of 1-(2-((tert-butyldimethylsilyl)oxy)ethyl)-3-(phenylethynyl)-1H-pyrazolo[3,4-d]pyrimidin-4-amine.

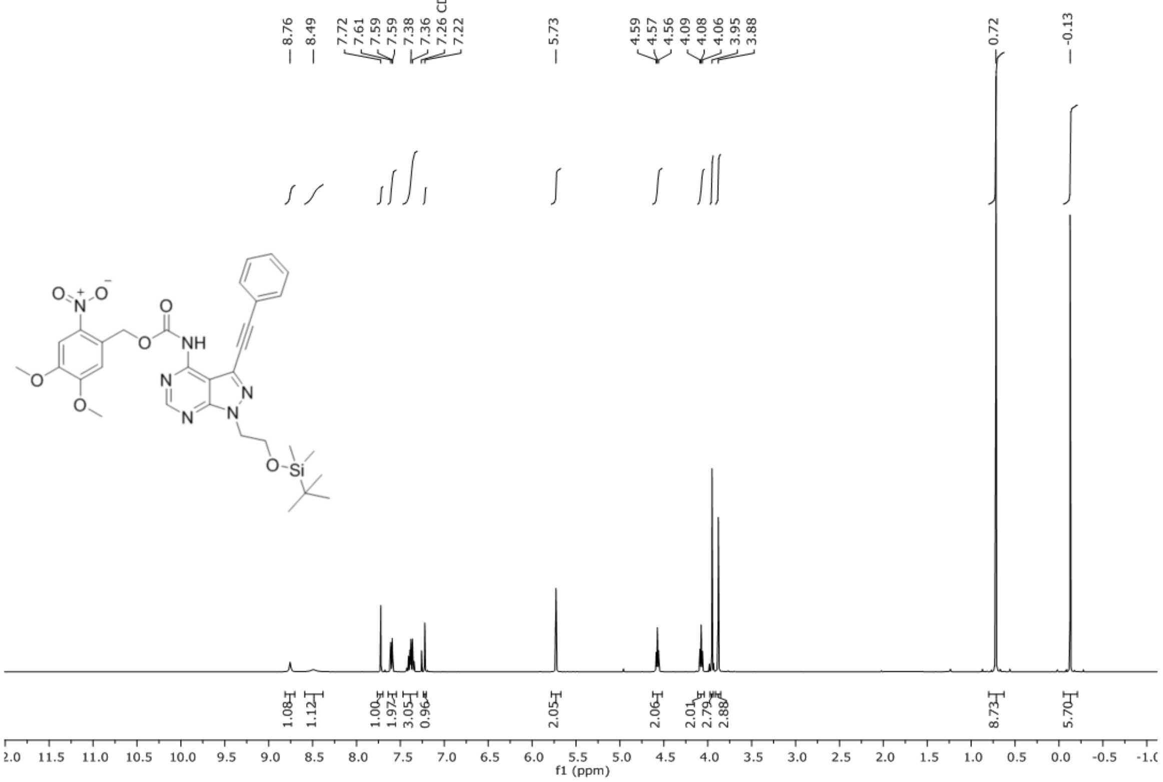


**Figure S11.** $^{1}$H-NMR (400 MHz, $CDCl_3$) spectra of 4,5-dimethoxy-2-nitrobenzyl-(1-(2-((tert-butyldimethylsilyl)oxy)ethyl)-3-(phenylethynyl)-1H-pyrazolo[3,4-d]pyrimidin-4-yl)carbamate.

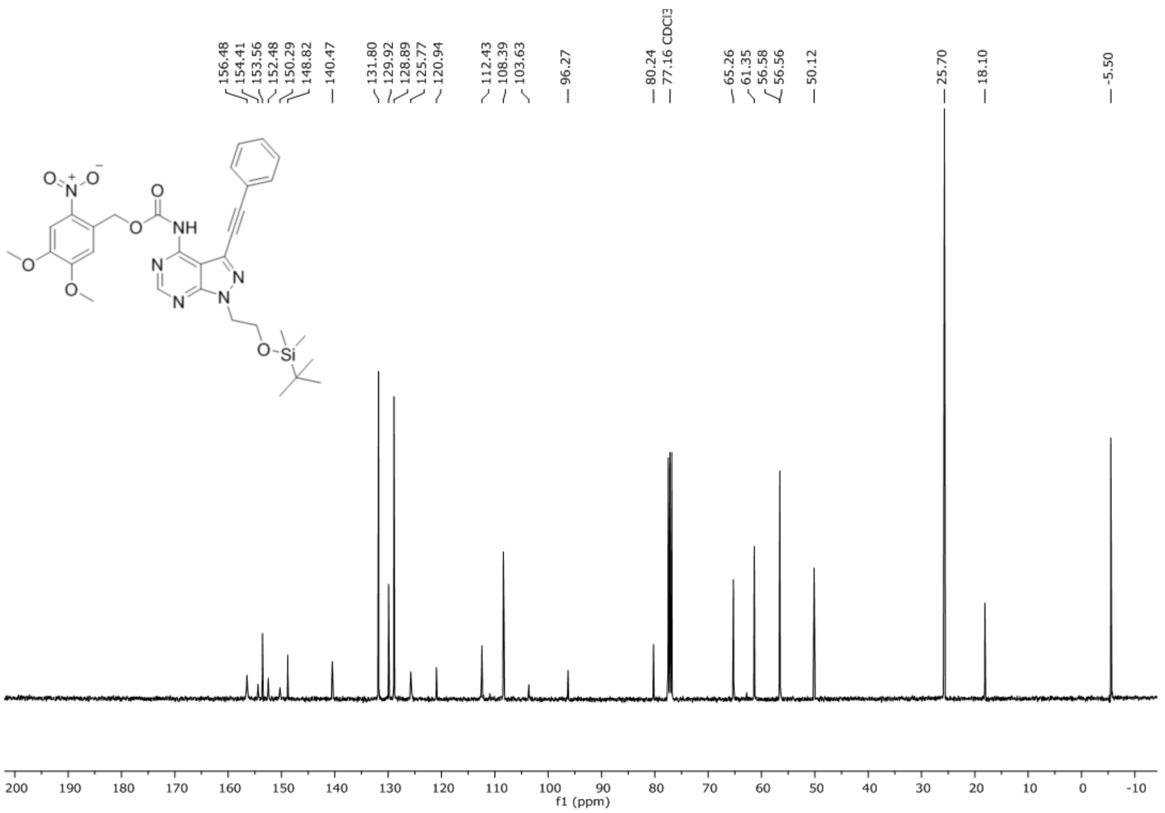


**Figure S12.** $^{13}$C-NMR (100 MHz, $CDCl_3$) spectra of 4,5-dimethoxy-2-nitrobenzyl-(1-(2-((tert-butyldimethylsilyl)oxy)ethyl)-3-(phenylethynyl)-1H-pyrazolo[3,4-d]pyrimidin-4-yl)carbamate.

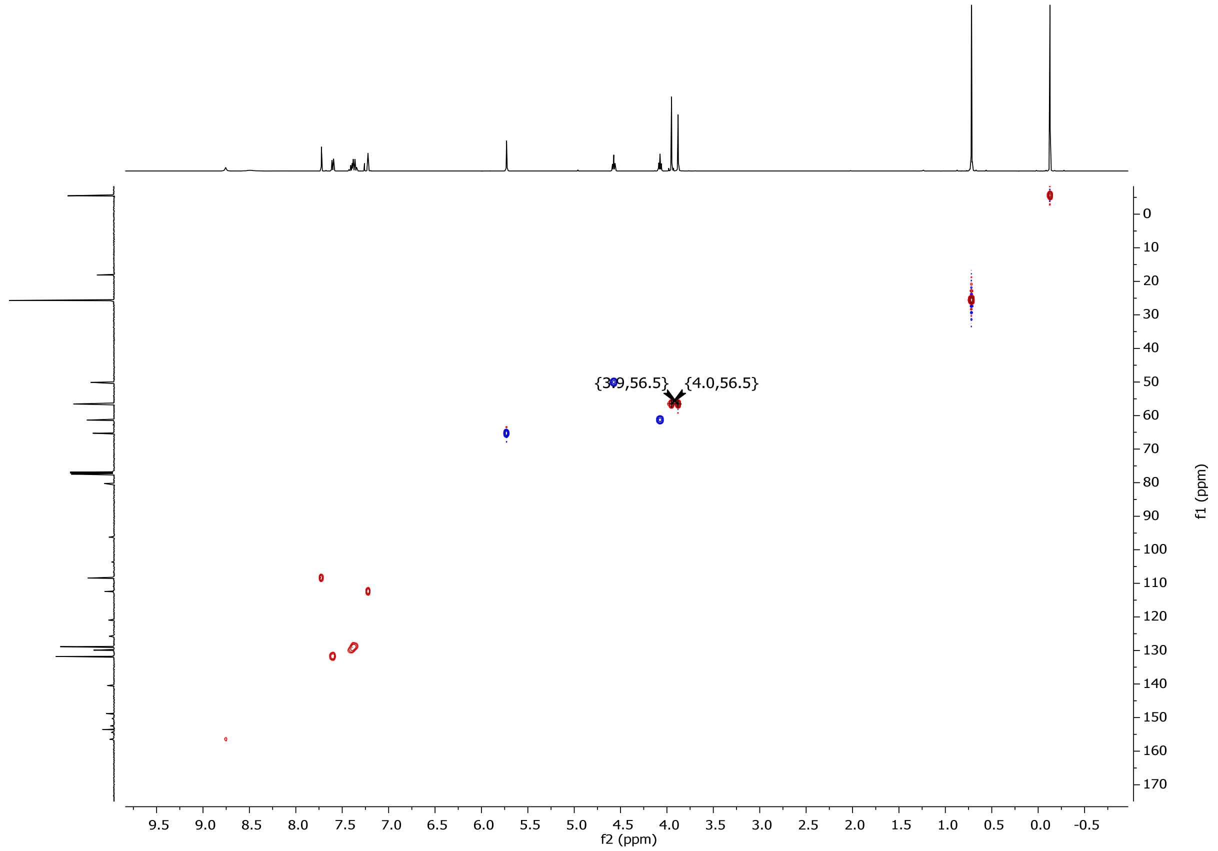


**Figure S13.** gHSQC of 4,5-dimethoxy-2-nitrobenzyl-(1-(2-((tert-butyldimethylsilyl)oxy)ethyl)-3-(phenylethynyl)-1H-pyrazolo[3,4-d]pyrimidin-4-yl)carbamate confirming the overlap of the methoxy carbon signals.

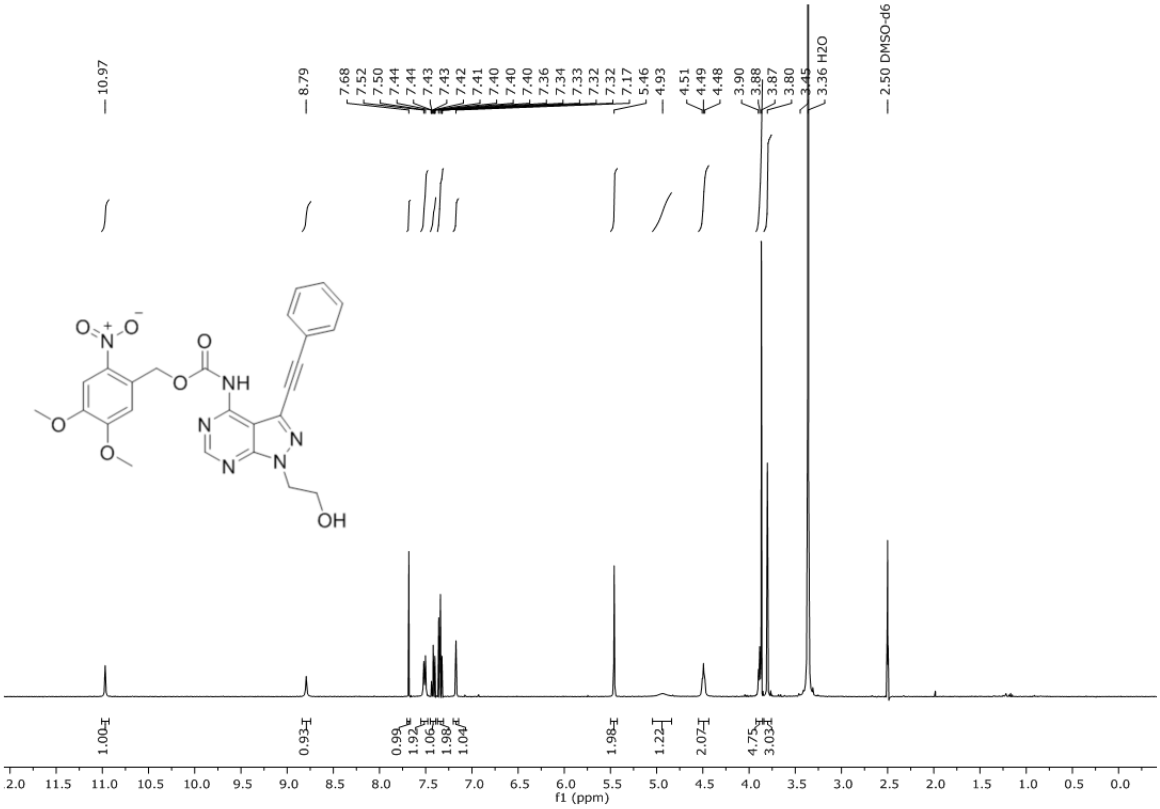


**Figure S14.** $^{1}$H-NMR (400 MHz, DMSO-$d_6$) spectra of **3**.

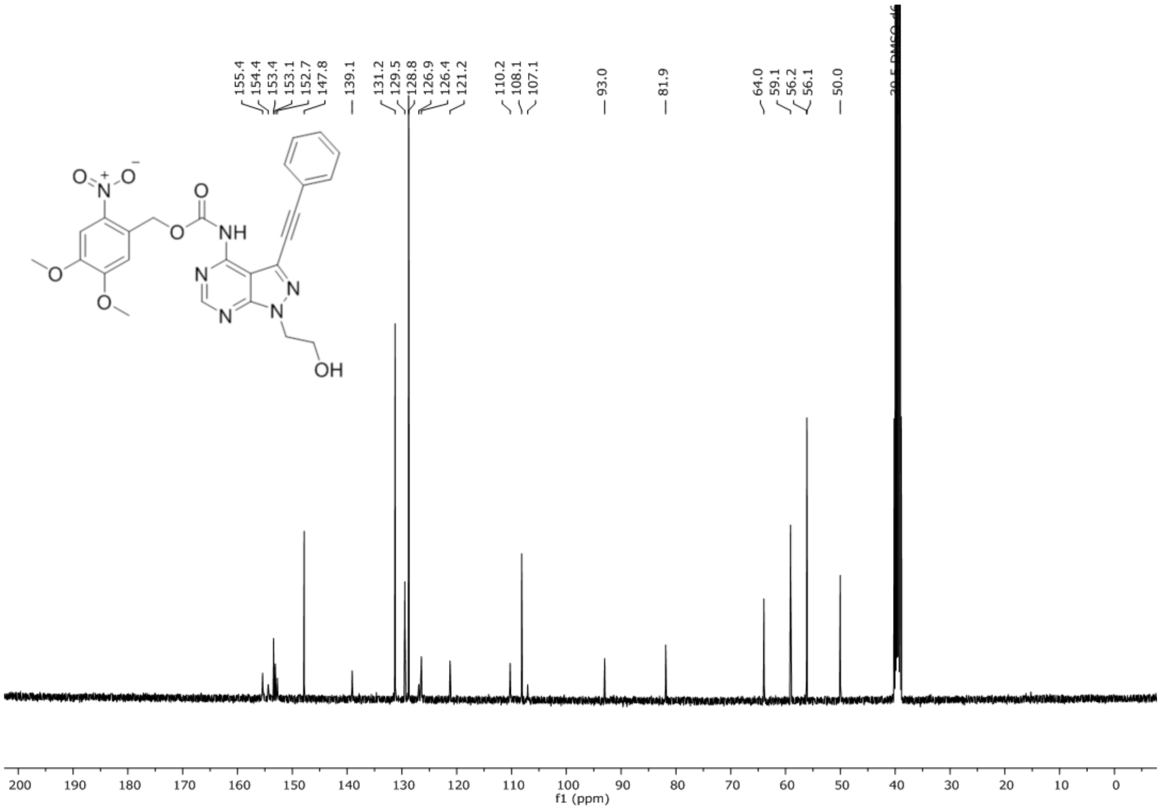


**Figure S15.** $^{13}$C-NMR (100 MHz, DMSO-$d_6$) spectra of **3**.

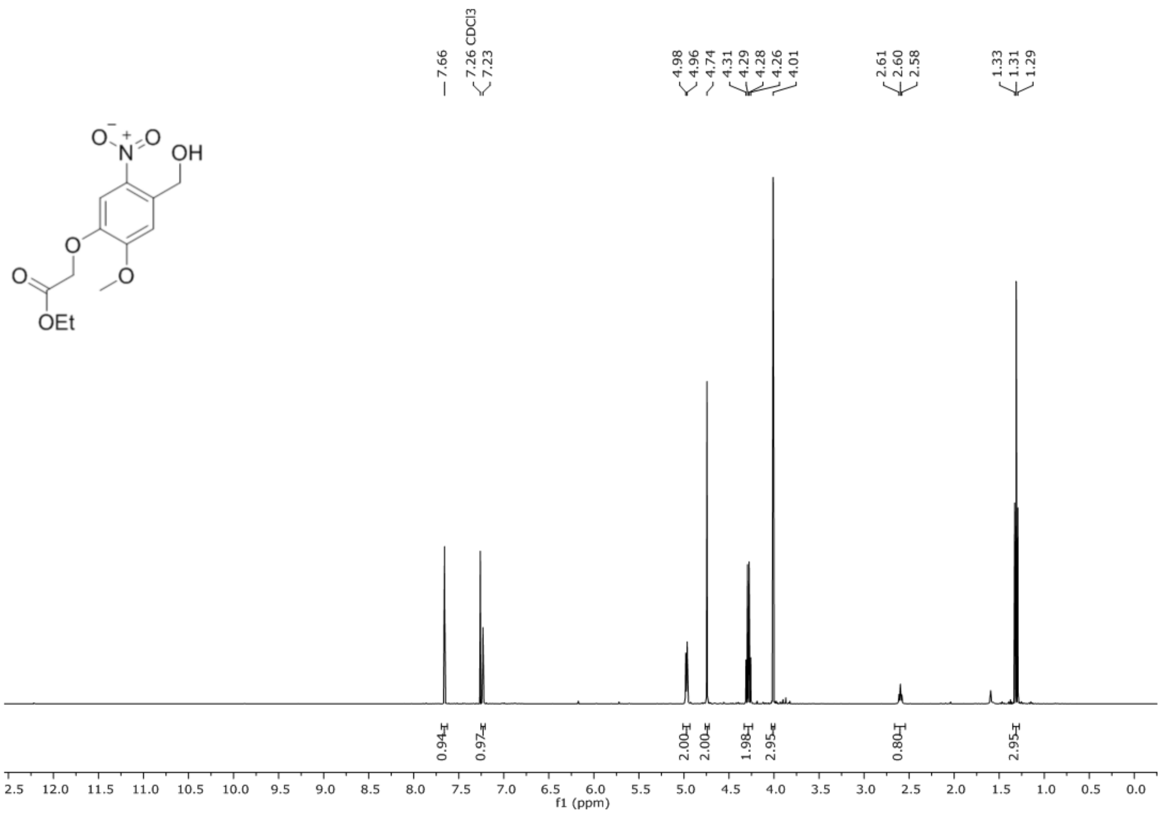


**Figure S16.** $^{1}$H-NMR (400 MHz, $CDCl_3$) spectra of **4**.

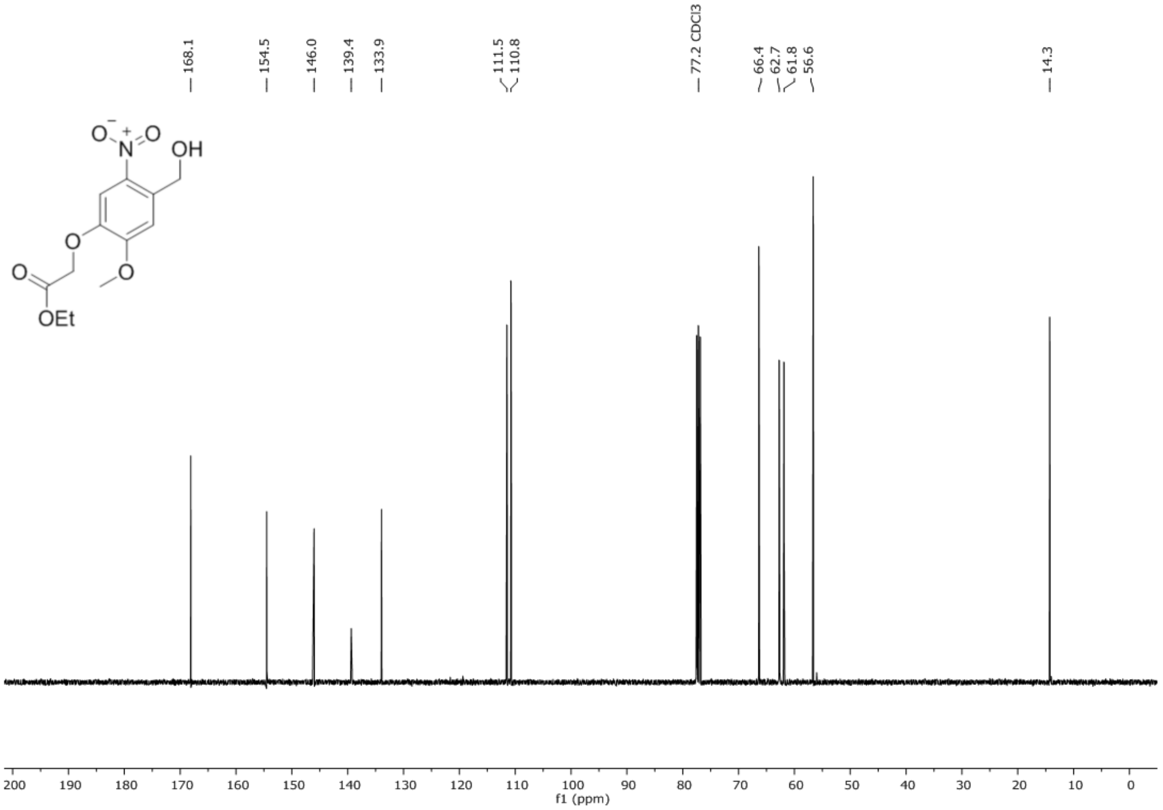


**Figure S17.** $^{13}$C-NMR (100 MHz, $CDCl_3$) spectra of **4**.

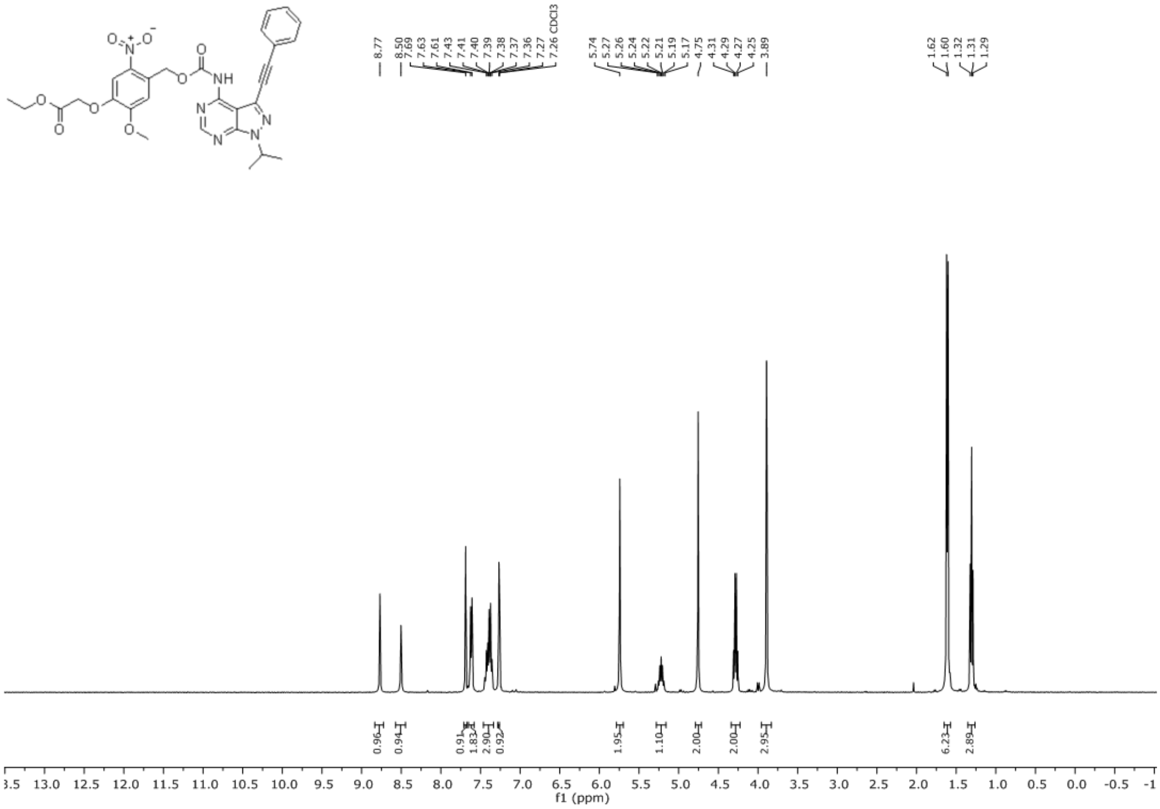


**Figure S18.** $^{1}$H-NMR (400 MHz, $CDCl_3$) spectra of **5**.

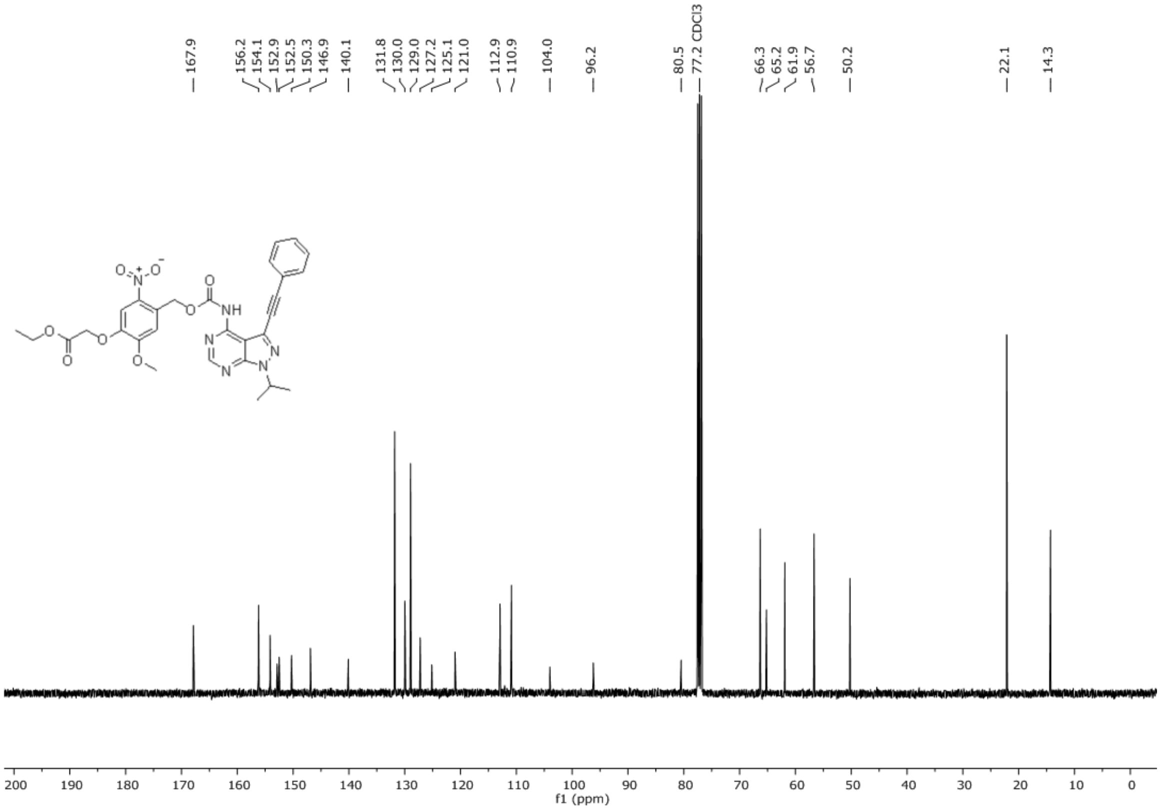


**Figure S19.** $^{13}$C-NMR (100 MHz, $CDCl_3$) spectra of **5**.

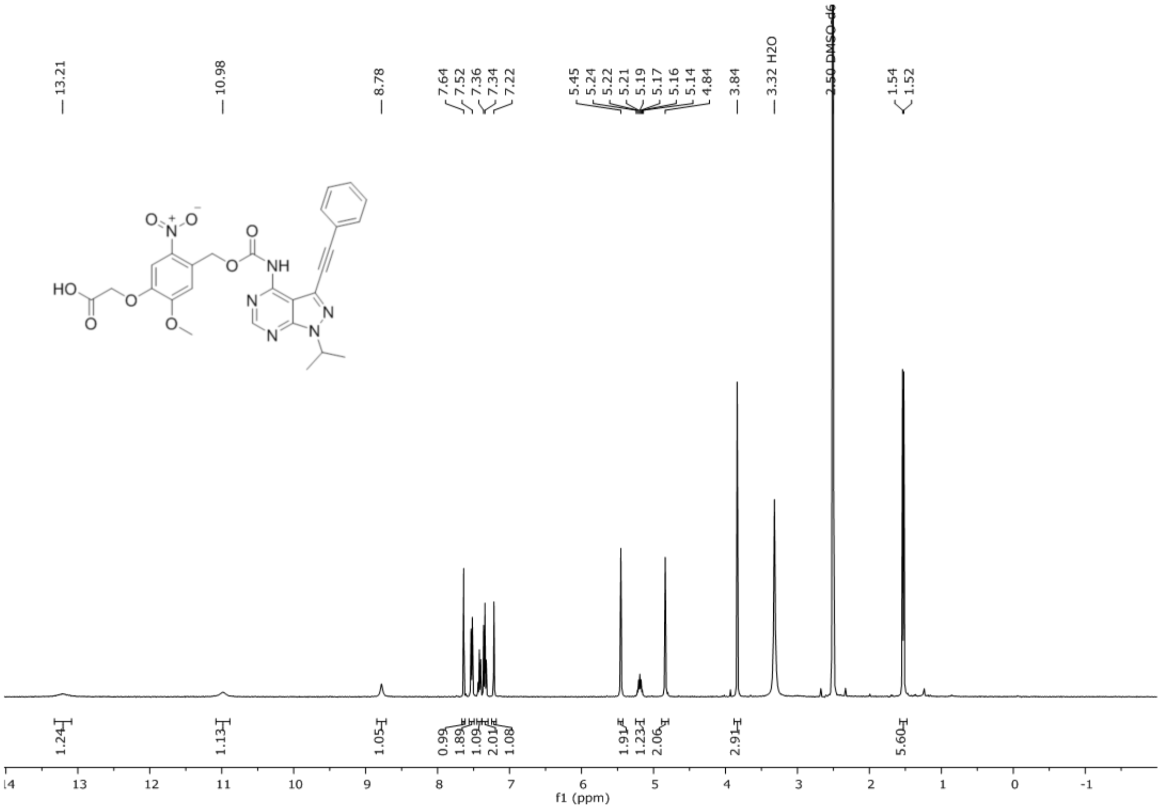


**Figure S20.** $^{1}$H-NMR (400 MHz, DMSO-$d_6$) spectra of **6**.

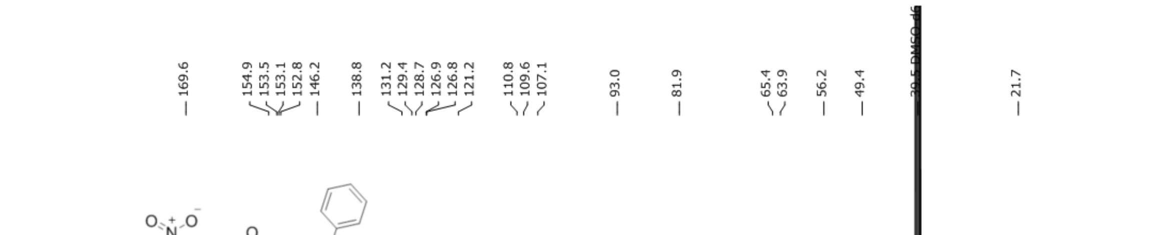


**Figure S21.** $^{13}$C-NMR (100 MHz, DMSO-$d_6$) spectra of **6**.

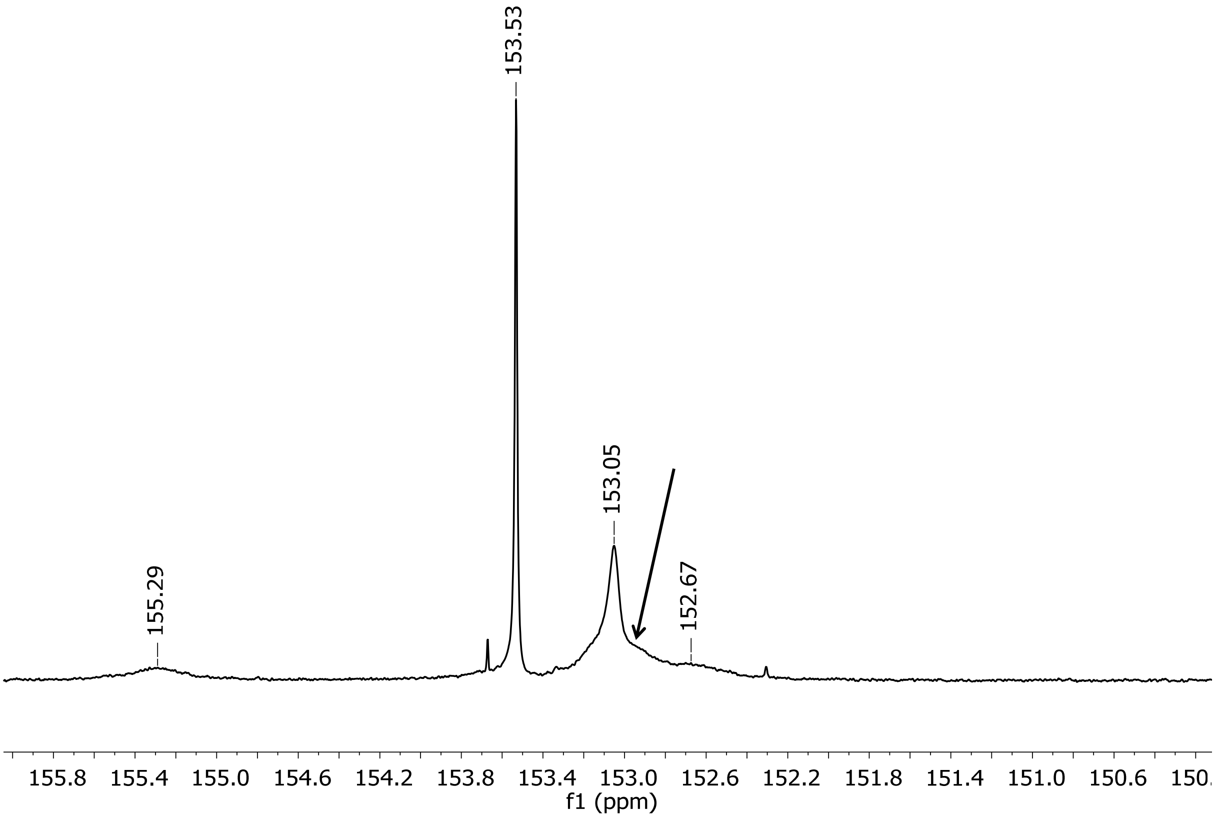


**Figure S22.** Detail of high field (800 MHz) $^{13}C$ NMR in DMSO-$d_6$ (0.6ml) and $CD_3OD$ (10µl) that reveals an overlap of two broad peaks (see arrow).

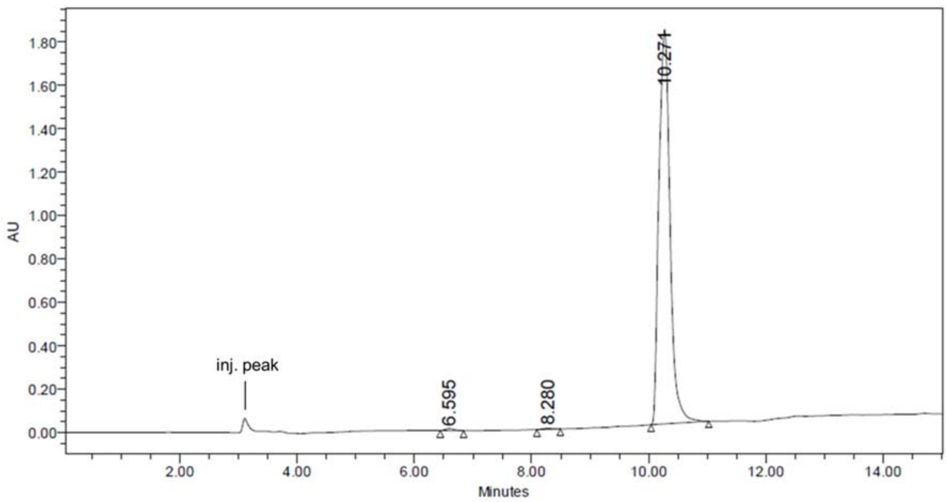


| | RT | Area | % Area | Height |
|---|---|---|---|---|
| 1 | 6.595 | 102265 | 0.43 | 9507 |
| 2 | 8.280 | 47384 | 0.20 | 3692 |
| 3 | 10.271 | 23869058 | 99.38 | 1820875 |

**Figure S23.** HPLC chromatogram of **6** at 240 nm (20-100% acetonitrile in water using 0.1% TFA as buffer).

## 7. References


1. Todorovic, N., Awuah, E., Shakya, T., Wright, G. D. & Capretta, A. Microwave-assisted synthesis of N1- and C3-substituted pyrazolo[3,4-d]pyrimidine libraries. *Tetrahedron Lett.* **52**, 5761-5763 (2011).
2. Klein, M. *et al.* Design, Synthesis and Characterization of a Highly Effective Inhibitor for Analog-Sensitive (as) Kinases. *PLoS ONE* **6**, e20789 (2011).
3. Dinér, P., Alao, J. P., Söderlund, J., Sunnerhagen, P. & Grøtli, M. Preparation of 3-Substituted-1-Isopropyl-1H-pyrazolo[3,4-d]pyrimidin-4-amines as RET Kinase Inhibitors. *J. Med. Chem.* **55**, 4872-4876 (2012).
4. Alvarez, K., Vasseur, J.-J., Beltran, T. & Imbach, J.-L. Photocleavable Protecting Groups as Nucleobase Protections Allowed the Solid-Phase Synthesis of Base-Sensitive SATE-Prooligonucleotides. *J. Org. Chem.* **64**, 6319-6328 (1999).
5. McMinn, D. L. & Greenberg, M. M. Novel solid phase synthesis supports for the preparation of oligonucleotides containing 3′-alkyl amines. *Tetrahedron* **52**, 3827-3840 (1996).
6. Molecular Operating Environment (MOE), 2012.10; Chemical Computing Group Inc., 1010 Sherbooke St. West, Suite #910, Montreal, QC, Canada, H3A 2R7, 2013.
